# Urine output is an early and strong predictor of acute kidney injury and associated mortality: a systematic literature review of 50 clinical studies

**DOI:** 10.1186/s13613-024-01342-x

**Published:** 2024-07-09

**Authors:** Manu L. N. G. Malbrain, Krista Tantakoun, Anthony T. Zara, Nicole C. Ferko, Timothy Kelly, Wojciech Dabrowski

**Affiliations:** 1 First Department of Anesthesiology and Intensive Therapy, Medical University of Lublin, Lublin, Poland; 2 International Fluid Academy, Lovenjoel, Belgium; 3 Medical Data Management, Medaman, Geel, Belgium; 4 Value & Evidence Division, Marketing and Market Access, EVERSANA™, Burlington, ON Canada; 5 Becton, Dickinson and Company, Franklin Lakes, NJ USA

**Keywords:** Acute kidney injury, Urine output, Serum creatinine, Detection, Prognosis, Systematic literature review, Epidemiology, AKIN, KDIGO, RIFLE

## Abstract

**Background:**

Although the present diagnosis of acute kidney injury (AKI) involves measurement of acute increases in serum creatinine (SC) and reduced urine output (UO), measurement of UO is underutilized for diagnosis of AKI in clinical practice. The purpose of this investigation was to conduct a systematic literature review of published studies that evaluate both UO and SC in the detection of AKI to better understand incidence, healthcare resource use, and mortality in relation to these diagnostic measures and how these outcomes may vary by population subtype.

**Methods:**

The systematic literature review was performed following the Preferred Reporting Items for Systematic Reviews and Meta-Analyses (PRISMA) checklist. Data were extracted from comparative studies focused on the diagnostic accuracy of UO and SC, relevant clinical outcomes, and resource usage. Quality and validity were assessed using the National Institute for Health and Care Excellence (NICE) single technology appraisal quality checklist for randomized controlled trials and the Newcastle–Ottawa Quality Assessment Scale for observational studies.

**Results:**

A total of 1729 publications were screened, with 50 studies eligible for inclusion. A majority of studies (76%) used the Kidney Disease: Improving Global Outcomes (KDIGO) criteria to classify AKI and focused on the comparison of UO alone versus SC alone, while few studies analyzed a diagnosis of AKI based on the presence of both UO and SC, or the presence of at least one of UO or SC indicators. Of the included studies, 33% analyzed patients treated for cardiovascular diseases and 30% analyzed patients treated in a general intensive care unit. The use of UO criteria was more often associated with increased incidence of AKI (36%), than was the application of SC criteria (21%), which was consistent across the subgroup analyses performed. Furthermore, the use of UO criteria was associated with an earlier diagnosis of AKI (2.4–46.0 h). Both diagnostic modalities accurately predicted risk of AKI-related mortality.

**Conclusions:**

Evidence suggests that the inclusion of UO criteria provides substantial diagnostic and prognostic value to the detection of AKI.

**Supplementary Information:**

The online version contains supplementary material available at 10.1186/s13613-024-01342-x.

## Background

As a subgroup of kidney disease, acute kidney injury (AKI) is defined by abnormalities in kidney function over six hours to one week and affects around 32% to 75% of intensive care unit (ICU) patients [1–8]. Notably, AKI has been associated with an adjusted incremental cost of over $8417 (2017 US dollars) and 2.9 days incremental length of hospital stay [9]. The pathophysiology of AKI in the ICU is multi-factorial, with the reported incidence varying between patient populations and history of comorbidities (e.g., cardiovascular disease, hemodynamic instability, infection, chronic kidney disease, liver disease, diabetes, surgery, and nephrotoxic drugs) [10].

Early detection of AKI can lead to sooner diagnosis and implementation of management techniques aimed at preventing or delaying the progression to increasingly severe disease [11]. Diagnosis of AKI can be assessed through multiple classification systems including the RIFLE (Risk, Injury, Failure, Loss of kidney function, and End-stage kidney disease), AKIN (Acute Kidney Injury Network), VARC-2 (Valve Academic Research Consortium-2), and KDIGO (Kidney Disease: Improving Global Outcomes) criteria, which all involve measurement of acute increases in serum creatinine (SC) and reduced urine output (UO) [12–14]. Most recently in 2012, the KDIGO organization released a newer classification, offering simplified and integrated diagnostic criteria [15, 16]. While diagnostic criteria have been standardized, variable assessment impedes researchers from comparing results and making conclusions about the precise impact of AKI care guidelines and bundles on patient outcomes [11].

Contributing to this assessment variability, UO is sometimes omitted in clinical practice as there are technical difficulties associated with accurate manual measurement at regular intervals, uploading values into an information system, and complexity in interpretation [17]. However, the value of UO measurement both alone or in combination with SC is reported to have the ability to detect AKI sooner and more accurately [18, 19], with intensive monitoring of UO having been associated with improved outcomes [20]. This suggests that there may be an overreliance on using SC as a trigger for AKI detection, which can result in missed or delayed diagnosis of approximately 20% of cases of AKI, potentially diagnosing the condition later than if UO were the trigger [8]. This is especially important in populations with fluid accumulation or hemodilution, where impaired UO can be detected without measurable increases in SC or adequate UO in patients with elevated SC without other symptoms of developing AKI [21–23].

Several studies have assessed the use of one or both of UO and SC to detect AKI across various subpopulations [24, 25]. However, the rates of AKI and associated outcomes substantially vary. As no systematic assessments of these data have been published, the potential explanation for these contradictory results is unclear. The purpose of the present investigation was to summarize all published studies that evaluate both UO and SC in the detection of AKI through a systematic literature review (SLR). This novel SLR will establish a baseline understanding of the incidence, healthcare resource use, morbidity, and mortality in relation to these diagnostic measures, and how these outcomes may vary by subgroups.

## Methods

### Search strategy

An a priori SLR protocol (unpublished) was developed that outlined the PICOS criteria (i.e., population, intervention, comparator, outcomes, and study design) and methodology (Additional file 1). This systematic literature review followed the Preferred Reporting for Systematic Reviews and Meta-Analyses (PRSIMA) guidelines (Additional file 2) [26]. The search strategy (Additional file 1) was developed by a medical information specialist in consultation with the review team, and peer reviewed prior to execution using the Peer Review of Electronic Search Strategies (PRESS) checklist [27]. Using the OVID platform, a systematic search of MEDLINE, EMBASE, and the Cochrane Central Register of Controlled Trials (CENTRAL) was conducted on July 6, 2022. Strategies utilized controlled vocabulary and keywords relevant to the research question (e.g., acute kidney injury, serum creatinine terms, urine output terms). Searches were limited by date from 2012 onwards to align with publication of recent KDIGO guidelines, and no language limits were applied. The search strategy aimed to locate appropriate research on UO as a marker of AKI, specifically focusing on comparative studies between UO and SC. Conference abstracts, posters, and narrative reviews were excluded. Reference lists of retrieved articles and relevant SLRs and meta-analyses were manually searched for additional studies.

### Study selection

Studies were selected for inclusion in the SLR based on pre-defined PICOS criteria. Studies that have a very small population, typically fewer than 20 patients, or that solely focus on infants or newborns have been excluded. Studies deemed eligible upon title and abstract screening were screened in full text using DistillerSR software (Ottawa, Ontario, Canada) [28]. Publications were reviewed in duplicate (KT and ATZ) at each stage and discrepancies were resolved by consensus, or by adjudication by a third reviewer (NF).

### Data extraction and outcomes

Baseline characteristics and outcomes from the included studies were extracted using a standardized extraction form developed in Microsoft Excel (Redmond, Washington, USA). Extracted details included study characteristics, population and baseline characteristics, results (e.g., time to diagnosis of AKI, incidence of AKI, mortality, health care resource use, follow-up period), and variables required for study quality assessments. Additional outcomes considered for extraction included, but were not limited to, the use of renal replacement therapy (RRT), organ failure-free days, vasopressor-free days, and ventilator-free days. In addition to specific diagnostic criteria used, deviations from these criteria were extracted (e.g., intensity and time interval for UO/SC collection, thresholds for AKI staging, etc.). Data were extracted by one reviewer (KT) and then examined for accuracy and completeness by a second reviewer (ATZ).

The outcome of focus was the incidence of AKI given the frequency of reporting and data availability by various subpopulations. Additional outcomes assessed were as follows: (1) timing to diagnosis of AKI, (2) adjusted mortality risk, and (3) hospital and ICU length of stay.

### Analysis

Acute kidney injury-related outcomes were evaluated according to the following diagnostic methods used to detect presence of AKI:

1. AKI_UO_ (i.e., positive on UO alone)
2. AKI_SC_ (i.e., positive on SC alone)
3. AKI_UO or SC_ (i.e., both criteria applied, patient positive on at least one)
4. AKI_UO and SC_ (i.e., both criteria applied, patient positive on both).

For incidence of AKI, box plots were generated describing the median and inter-quartile range (IQR) across studies. Data were presented according to subgroups to assess how incidence rates varied. Data were stratified according to UO measurement frequency [i.e., intensive measurement (hourly recordings) versus less intensive (recordings occurred more than one hour apart)], age [i.e., > 60 versus ≤ 60 years and adult versus pediatric patients (excluding infants and newborns)], and cardiac versus non-cardiac patients. Given the fluid overload and accumulation implications [29], another subgroup analysis was performed on cardiovascular-focused studies that enrolled cardiopulmonary bypass (CPB) patients versus those that did not [30].

Studies that reported timing of diagnosis of AKI after admission were qualitatively summarized. Hospital and ICU length of stay were reported by study according to diagnostic method; results from studies were typically unadjusted. Lastly, mortality related to AKI was explored by generating forest plots of adjusted odds ratios (ORs) or hazard ratios (HRs) and 95% confidence intervals (CIs) were extracted from studies reporting adjusted mortality risk between those with and without diagnosed AKI; studies were not pooled due to substantial heterogeneity in population characteristics and timepoints for mortality assessments, but rather presented individually.

In addition, meta-analyses were not performed given the substantial heterogeneity across studies in terms of patient population and reporting of outcomes.

### Quality assessment

The quality of studies included in the SLR was assessed using the National Institute for Health and Care Excellence (NICE) single technology appraisal quality checklist for randomized controlled trials (RCTs) and the Newcastle–Ottawa Quality Assessment Scale (NOS) for observational studies [31, 32]. Modified versions of the scale were also considered, and total scores were converted to an eight-point scale. The quality of included studies was assessed independently by two reviewers (KT, ATZ) and reconciled by a third reviewer (NF), if required.

## Results

**Fig. 1:**
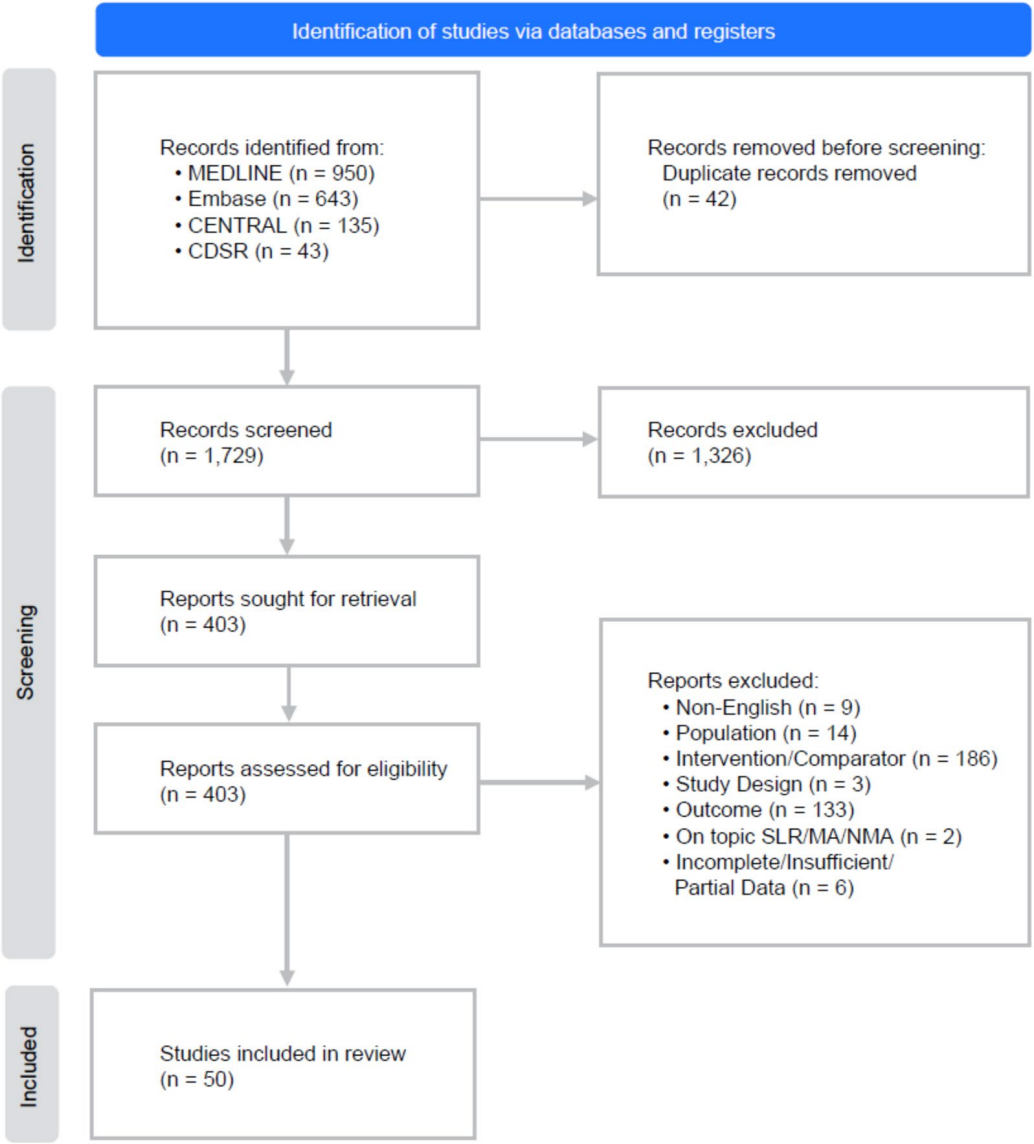
PRISMA flow diagram. *CDSR* Cochrane Database of Systematic Reviews, *MA* meta-analysis, *NMA* network meta-analysis, *SLR* systematic literature review

A total of 1771 citations were identified from searches. After removing duplicates, 1729 unique records were screened. Of those, 1326 were excluded for various reasons (e.g., non-human, noncomparative) (Fig. 1). One RCT and 49 observational studies were included in the SLR.

Study characteristics are presented in Table 1. The sample sizes of the 50 included studies ranged from 57 in a single center observational cohort study to 155,624 in a retrospective database analysis. Most studies (76%) used the KDIGO criteria to classify AKI and focused on the comparison of UO alone with SC alone; however, a few studies also included comparisons with UO and SC (AKI_UO and SC_ criteria), and UO or SC (AKI_UO or SC_ criteria). A third of the included studies enrolled patients with cardiovascular-related conditions (33%). Of these, four studies (7%) enrolled patients undergoing cardiac surgery with CPB and one study (2%) enrolled patients undergoing cardiac surgery with or without CPB. Another third enrolled all patients admitted to the general ICU (30%). The remaining third was comprised of studies that enrolled pediatric patients (11%), liver-related conditions (7%), kidney-related conditions (7%), non-cardiac surgery (4%), orthopedic surgery (2%), cancer (2%), parasitic infection (2%), and COVID-19 (2%). The study follow-up durations ranged from 48 h to over 5 years.

**Table 1:** Study characteristics for studies included in the SLR

Study Reference	Study Design	Population^a^	Sample Size	AKI Diagnostic Criteria(s) Used	Measure(s) Used	Follow-up Duration	Outcomes^b^
Prospective studies							
Bouchard et al. [48]	Observational cohort study	General ICU	80	AKIN^c^	UO, SC	≤ 10 days	Incidence & staging, timing of AKI, hospital LOS
Chau et al. [66]	Observational cohort study	Cardiovascular	111	AKIN^c,d^	UO, SC	72 h	Incidence & staging, hospital LOS
Goldani et al. [71]	Observational cohort study	Cardiovascular	114	KDIGO^c,d^	UO, SC, UO or SC	24 h	Incidence & staging, other diagnostic outcomes
Howitt et al. [56]	Observational cohort study	Cardiovascular	2267	KDIGO	UO, SC, UO and SC	2 years	Incidence & staging, survival, HCRU
Kaddourah et al. [72]	Observational cohort study	Pediatric	3318	KDIGO^c,d^	UO, SC, UO and SC	≤ 28 days	Incidence & staging, survival, ICU LOS
Kellum et al. [8]	Observational cohort study	General ICU	32,045	KDIGO^c^	UO, SC, UO or SC	≤ 1 year	Incidence & staging, survival, ICU LOS, hospital LOS
Koeze et al. [49]	Observational cohort study	General ICU	361	KDIGO	UO, SC	48 h	Incidence & staging, survival, ICU LOS
Luther et al. [69]	Observational cohort study	COVID-19	57	KDIGO	UO, SC	30 days	Incidence & staging, timing of AKI
McCullough et al. [33]	RCT	Cardiovascular	231	AKIN, RIFLE, KDIGO^c,d^	UO or SC	120 days	Incidence & staging, timing of AKI, ICU LOS, hospital LOS
Md Ralib et al. [65]	Audit	General ICU	725	KDIGO	UO, SC	12 months	Incidence & staging, other diagnostic outcomes, survival
Oshomah-Bello et al. [54]	Observational cohort study	Pediatric; Parasitic Infection	244	KDIGO^c^	UO, SC, UO or SC	NR	Incidence & staging, survival
Palermo et al. [73]	Observational pilot cohort study	Pediatric	81	KDIGO^c^	SC, UO or SC	14 days	Incidence & staging, survival, HCRU
Petäjä et al. [59]	Observational cohort study	Cardiovascular	638	KDIGO	UO, SC, UO and SC	~ 2.5 years	Incidence & staging, timing of AKI, survival, ICU LOS
Tarvasmaki et al. [47]	Observational cohort study	Cardiovascular	154	KDIGO^c^	UO, SC	≤ 90 days	Incidence & staging, timing of AKI, survival
Vandenberghe et al. [78]	Observational cohort study	Cardiovascular	100	KDIGO^c,d^	UO, SC	48 h	Incidence & staging, other diagnostic outcomes
Wiersema et al. [70]	Observational cohort study	General ICU	1010	KDIGO	UO, SC, UO or SC	90 days	Incidence & staging, survival
Willner et al. [24]	Observational cohort study	Kidney	95	KDIGO	UO, SC, UO or SC	1 year	Incidence & staging, timing of AKI, ICU LOS, hospital LOS
Wlodzimirow et al. [76]	Observational cohort study	General ICU	260	RIFLE	UO, SC, UO and SC	NR	Incidence & staging, timing of AKI, other diagnostic outcomes, survival
Retrospective studies							
Allen et al. [38]	Analysis of observational studies	General ICU; Cardiovascular	301	KDIGO^c,e^	UO, SC, UO or SC	1 year	Incidence & staging, other diagnostic outcomes, survival
Amathieu et al. [40]	Observational cohort study	Liver	3458	KDIGO^c,d^	UO, SC, UO or SC	1 year	Incidence & staging, other diagnostic outcomes, survival
Bianchi et al. [51]	Observational cohort study	General ICU	15,620	KDIGO	UO, SC, UO or SC	Median:67 months	Incidence & staging, other diagnostic outcomes, survival, ICU LOS, hospital LOS
Bressan et al. [39]	Observational cohort study	Liver	80	AKIN^c^	UO, SC	30 days	Incidence & staging, survival, hospital LOS
Cordova-Sanchez et al. [42]	Observational cohort study	Oncology	389	KDIGO^c^	UO, SC, UO or SC	180 days	Incidence & staging, survival, ICU LOS
D’Arienzo et al. [36]	Observational cohort study	Pediatric; Cardiovascular; Non-cardiac Surgery	2051	KDIGO^c^	UO (N = 964), SC (N = 2003), UO or SC (N = 2051)	≥ 5 years	Incidence & staging, other diagnostic outcomes, survival
Engoren et al. [41]	Observational cohort study	Cardiovascular	4195	SC: KDIGO UO: KDIGO, RIFLE, AKIN^d^	UO, SC	72 h	Incidence & staging, other diagnostic outcomes, survival, ICU LOS, hospital LOS
Han et al. [46]	Observational cohort study	General ICU	1625	AKIN^d^	UO, SC, UO and SC	3 years	Incidence & staging, timing of AKI, other diagnostic outcomes, survival
Harris et al. [44]	Database analysis	General ICU	155,624	RIFLE^c,d^	UO, SC, UO or SC	NR	Survival, ICU LOS, hospital LOS
Hessey et al. [58]	Observational cohort study	Pediatric	2041	KDIGO^c^	SC (N = 1575), UO or SC (N = 1622)	5–7 years	Incidence & staging, other diagnostic outcomes, survival, ICU LOS, hospital LOS
Hessey et al. [57]	Observational cohort study	Pediatric	2041	KDIGO^c,d^	SC (N = 1575), UO or SC (N = 1622)	5 years	Incidence & staging, ICU LOS, hospital LOS
Hocine et al. [74]	Observational cohort study	Cardiovascular	149	CA-AKI, RIFLE^c^	UO, SC, UO and SC	3 days	Incidence & staging, timing of AKI, other diagnostic outcomes, survival, ICU LOS
Jiang et al. [50]	Observational cohort study	Kidney	14,725	KDIGO^d,f^	UO, SC, UO or SC	NR	Incidence & staging, other diagnostic outcomes, survival, ICU LOS
Jin et al. [20]	Observational cohort study	General ICU	15,724	KDIGO	UO, SC	30 days	Incidence & staging, survival, HCRU
Joliat et al. [55]	Observational cohort study	Liver	285	KDIGO^c,d^	UO, SC	30 days	Incidence & staging, survival, hospital LOS
Katabi et al. [68]	Observational cohort study	Cardiovascular	141	KDIGO^c,d^	UO, SC	6–12 months	Incidence & staging, other diagnostic outcomes, survival, ICU LOS, hospital LOS
Koeze et al. [4]	Observational cohort study	General ICU	1376	RIFLE, AKIN, KDIGO	UO, SC, UO or SC	7 days	Incidence & staging, timing of AKI
Lagny et al. [63]	Observational cohort study	Cardiovascular	443	RIFLE^c,d^	UO, SC	1 year	Incidence & staging, survival, ICU LOS, hospital LOS
Leite et al. [34]	Analysis of a prospective cohort study	Kidney	150	AKIN^c,d^	UO, SC, UO or SC	NR	Incidence & staging, survival, ICU LOS
McIlroy et al. [45]	Observational cohort study	Cardiovascular	311	AKIN	UO, SC, UO and SC	48 h	Incidence & staging, survival, ICU LOS, hospital LOS
Mizota et al. [43]	Secondary analysis of prospective data	Liver	320	KDIGO^c^	UO, SC, UO and SC	90 days	Incidence & staging, survival, ICU LOS, hospital LOS
Nikkinen et al. [52]	Registry analysis	Orthopedic Surgery	901	KDIGO^c,f^	UO, SC, UO and SC	1 year	Incidence & staging, timing of AKI, survival
Priyanka et al. [53]	Observational cohort study	Cardiovascular	6637	KDIGO^c^	UO, SC	180 days	Incidence & staging, survival
Qin et al. [61]	Observational cohort study	General ICU	1058	KDIGO^c,d,e^	UO, SC (N = 1058)	Until discharge	Incidence & staging, other diagnostic outcomes, survival
Quan et al. [60]	Observational cohort study	Non-cardiac Surgery	4229	KDIGO^c,d^	UO, SC, UO or SC	30 days	Incidence & staging, other diagnostic outcomes, survival, hospital LOS
Shacham et al. [75]	Observational cohort study	Cardiovascular	143	VARC-2	UO, SC	30 days	Incidence & staging, timing of AKI
Sims et al. [77]	Observational cohort study	Cardiovascular	5701	AKIN^c,d^	UO, SC	30 days	Incidence & staging, other diagnostic outcomes
Törnblom et al. [67]	Post-hoc analysis	General ICU	2044	KDIGO	UO, SC, UO or SC	< 90 days	Incidence & staging, other diagnostic outcomes, survival, ICU LOS
Tujjar et al. [64]	Observational cohort study	Cardiovascular	199	Definition aligns with KDIGO^c^	UO, SC, UO and SC	NR	Incidence & staging
Tulgar et al. [35]	Observational cohort study	Kidney	70	KDIGO^c,d^	UO, SC	NR	Incidence & staging, timing of AKI, survival
Vaara et al. [62]	Secondary analysis of prospective data	General ICU	2160	KDIGO^d^	UO, SC, UO and SC	≤ 90 days	Incidence & staging, timing of AKI, survival, ICU LOS
Vanmassenhove et al. [37]	Observational cohort study	General ICU	13,403	KDIGO^c,e^	UO, UO or SC	NR	Incidence & staging, other diagnostic outcomes, survival

*AKI* acute kidney injury, *AKIN* Acute Kidney Injury Network, *CA-AKI* contrast-associated acute kidney injury, *COVID-19* coronavirus disease 2019, *ICU* intensive care unit, *KDIGO* Kidney Disease: Improving Global Outcomes, *LOS* length of stay, *NR* not reported, *RCT* = randomized controlled trial, *RIFLE* risk, injury, failure, loss, and end-stage kidney disease, *SC* serum creatinine; SLR = systematic literature review, *UK* United Kingdom, *UO* urine output, *USA* United States of America

^a^Unless otherwise indicated, populations were adult

^b^Other diagnostic outcomes included reclassification of AKI staging and accuracy outcomes, including sensitivity and specificity of AKI detection. Survival outcomes include mortality rate, and/or mortality risk

^c^Study either did not adhere to hourly measurement of UO or did not report frequency of UO collection

^d^Study either did not adhere to at least daily measurement of SC or did not report frequency of SC collection

^e^Multiple definitions of UO criteria were evaluated

^f^The timeframe over which UO threshold was averaged differed from the recommended 6–12 h

### Quality assessment

Risk of bias and study quality assessments for the single RCT included are presented in Additional file 3 [33]. The quality of the study by McCullough et al. was acceptable as it had low or unclear risk of bias across all domains.

The NOS assessments for observational studies are presented in Additional file 4, with scores that ranged from six to eight stars. Most studies included patients who were either truly or somewhat representative of the exposed cohort. Two retrospective studies selected patients who were on RRT and were not given a star due to selection bias [34, 35]. All studies drew the cohorts from the same community, used secure surgical records for the ascertainment of exposure, used links to surgical records to assess the outcomes, and had follow-up durations that were long enough for outcomes to occur. All studies had either complete follow-up of all patients or some loss to follow-up unlikely to bias results (i.e., less than 20%). The studies varied most regarding the comparability of the cohorts. One star was given to studies that controlled for select demographic characteristics [8, 20, 24, 36–48], whereas two stars were given to studies that controlled for several confounders using regression analyses [34, 49–66]. Some studies only reported univariate or unadjusted results and no regression analyses showing lack of effect on outcomes and were not given a star due to potential confounding bias [4, 35, 67–78].

### Incidence of AKI

**Fig. 2:**
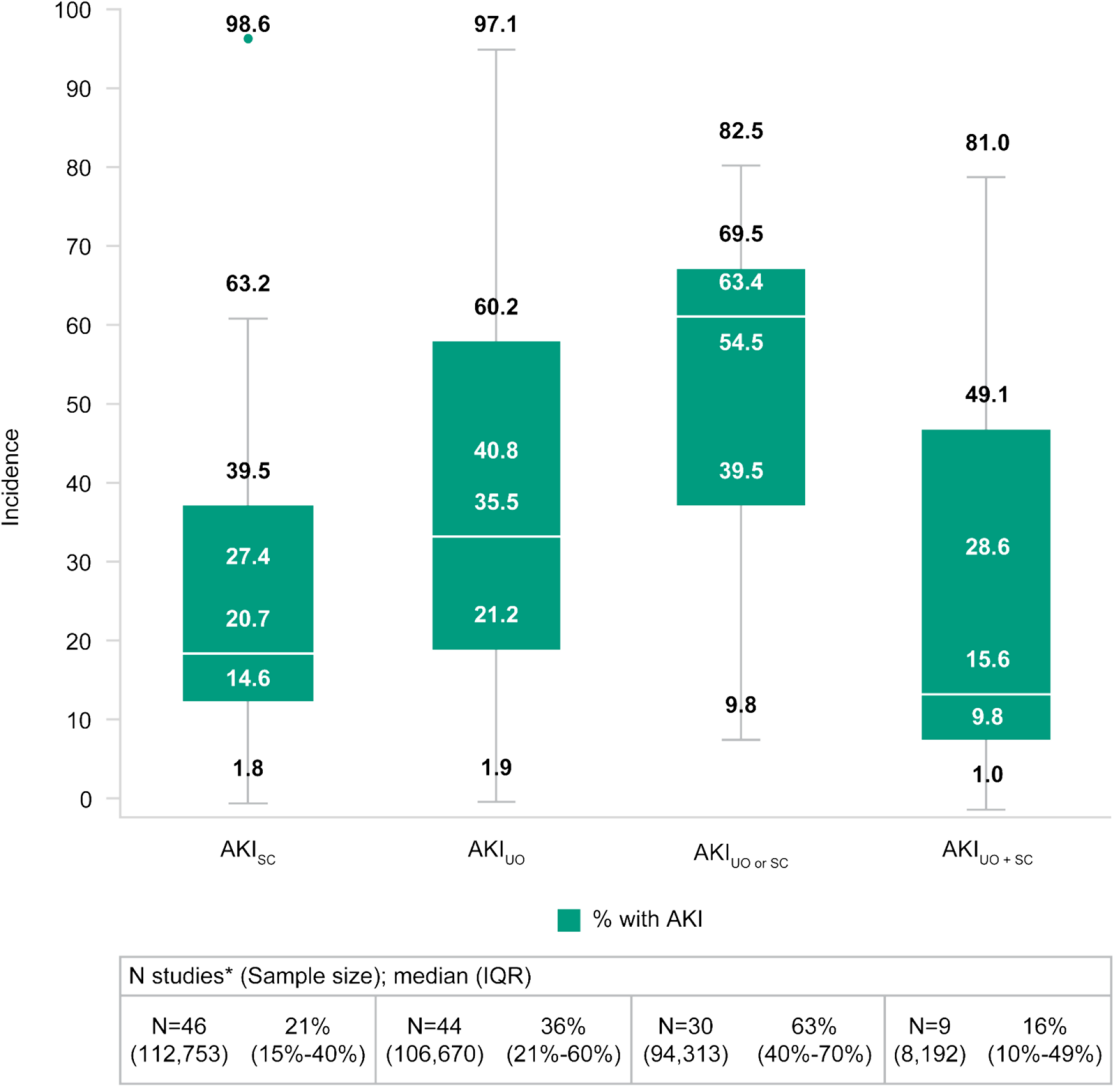
Incidence of AKI—All patient populations. *Some studies contributed multiple data points for a given diagnostic method. Boxplots compare AKI incidence based on diagnostic criteria used. The error bars are the range excluding outliers. The bottom and top of the box are the 25th and 75th percentiles, and the line inside the box is the 50th percentile (median). *AKI* acute kidney injury, *AKI*_*SC*_ AKI-positive according to SC criteria alone, *AKI*_*UO*_ AKI-positive according to UO criteria alone, *AKI*_*UOandSC*_ AKI-positive according to both UO and SC criteria—both tests collected, *AKI*_*UOorSC*_ AKI-positive according to UO criteria SC criteria, or both—both tests collected, *IQR* interquartile range

**Fig. 3:**
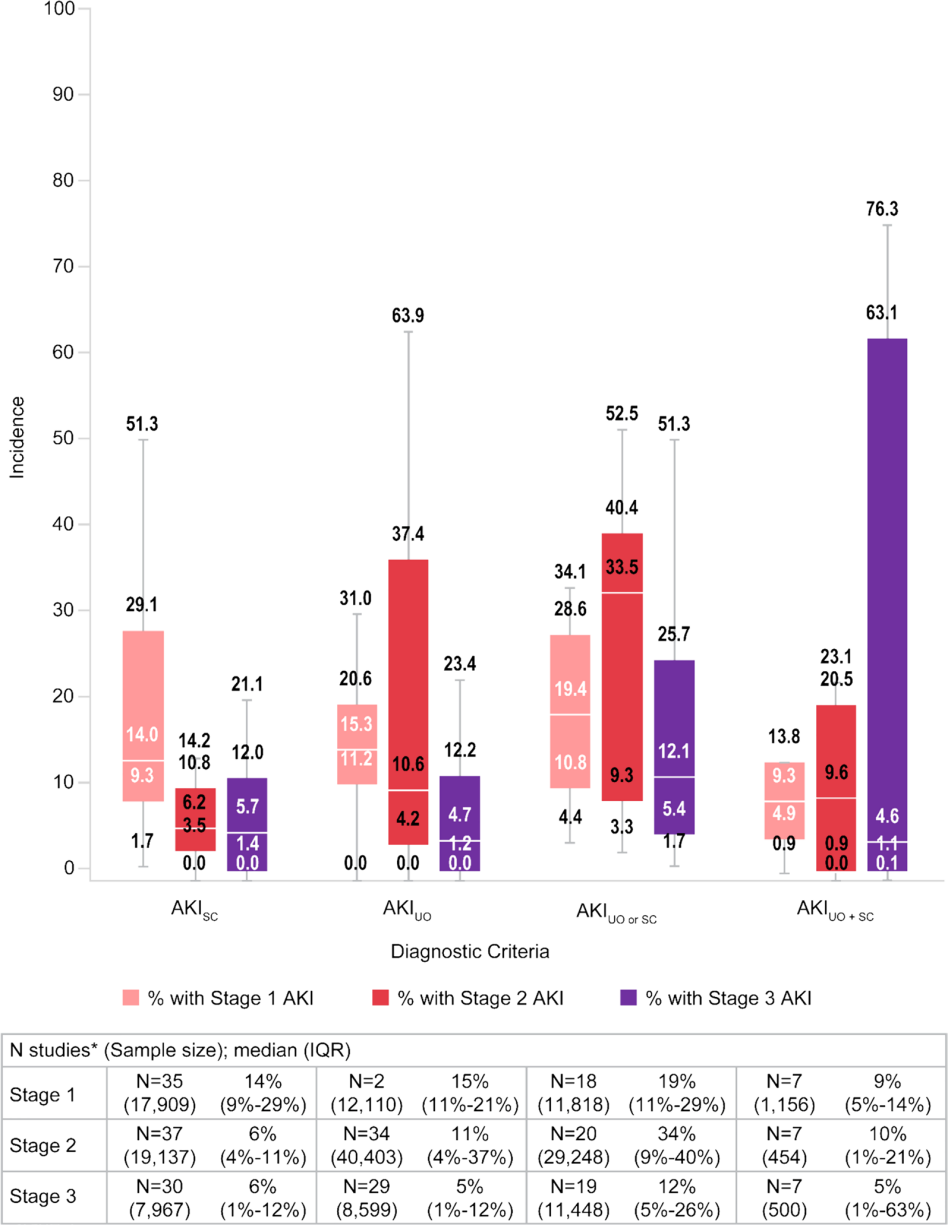
Incidence of AKI by staging – All patient populations. *Some studies contributed multiple data points for a given diagnostic method. Boxplots comparing AKI incidence based on diagnostic criteria used. The error bars are the range excluding outliers, the bottom and top of the box are the 25th and 75th percentiles, the line inside the box is the 50th percentile (median). *AKI* acute kidney injury, *AKI*_*SC*_ AKI-positive according to SC criteria alone, *AKI*_*UO*_ AKI-positive according to UO criteria alone, *AKI*_*UOandSC*_ AKI-positive according to both UO and SC criteria—both tests collected, *AKI*_*UOorSC*_ AKI-positive according to UO criteria SC criteria, or both—both tests collected, *IQR* interquartile range

The proportion of patients diagnosed with AKI was reported in all the included studies, except one in which the incidence of AKI was displayed as a heatmap (data could not be reliably extracted) [44]. The SLR results demonstrated that the incidence of AKI was highest among studies that used AKI_UO or SC_ criteria (median, IQR: 63%, 40%–70%) followed by those that used AKI_UO_ only (median, IQR: 36%, 21%–60%; Fig. 2). The incidence of AKI by staging is displayed in Fig. 3. It was observed that pooled rates of moderate to severe AKI staging (i.e., stages 2 and 3) were highest with the most sensitive criteria of AKI_UO or SC_.

#### Subgroup analyses

These findings remain consistent across the various patient subgroups (Table 2). Interestingly, more intensive UO monitoring (defined as repeat UO monitoring at least once every hour) was associated with almost twice (56% versus 31%) the incidence of AKI diagnosis compared with less intensive UO monitoring (defined as delay in between UO measurements spaced at least one hour apart). Irrespective of subgroup, UO alone tended to be associated with a higher incidence of AKI compared to the use of SC alone. The incidence of AKI appeared to be greatest in those patients undergoing CPB and was twice the rate (61% versus 31%) of those without CPB as measured by UO criteria alone. While it was expected that the incidence of AKI_UO or SC_ would be highest compared to the other diagnostic methods, there were some subgroups where AKI_UO_ or AKI_UO and SC_ were greater. For example, the ≤ 60-year-old subgroup incidence by AKI_UO_ was greater than AKI_UO or SC_ (34% vs. 23%), which may be a product of low study and patient numbers for this subgroup.

**Table 2:** Incidence of AKI—subgroup analyses

	N studies^a^ (sample size); median (IQR)							
	AKI_SC_		AKI_UO_		AKI_UO or SC_		AKI_UO and SC_	
UO measurement frequency								
Intensive (≤ 1 h)	N/A	N/A	N = 11 (27,935)	56% (26%–64%)	N = 6 (22,885)	41% (17%–68%)	N = 2 (2471)	38% (26%–50%)
Less intensive (> 1 h)	N/A	N/A	N = 18 (29,184)	31% (23%–54%)	N = 17 (25,416)	63% (46%–69%)	N = 4 (4992)	16% (10%–32%)
Stratified by age^b^								
Age ≤ 60	N = 13 (7,104)	21% (20%–38%)	N = 10 (2941)	34% (28%–55%)	N = 6 (4613)	23% (18%–41%)	N = 1 (260)	81%
Age > 60	N = 17 (30,237)	20% (13%–39%)	N = 20 (30,237)	35% (20%–63%)	N = 18 (19,234)	66% (53%–70%)	N = 4 (4402)	15% (10%–30%)
Cardiac versus non-cardiac								
Cardiac AKI	N = 13 (14,354)	18% (10%–20%)	N = 14 (14,354)	45% (23%–67%)	N = 8 (4691)	66% (57%–69%)	N = 4 (2926)	26% (14%–43%)
Non-cardiac AKI	N = 46 (115,062)	26% (15%–46%)	N = 44 (106,670)	33% (23%–56%)	N = 30 (94,313)	58% (40%–69%)	N = 9 (8192)	14% (10%–28%)
CPB versus non-CPB								
Cardiac AKI with CPB	N = 4 (7505)	19% (16%–25%)	N = 4 (7505)	61% (52%–69%)	N = 1 (114)	69%	N = 1 (311)	62%
Cardiac AKI, non-CPB	N = 9 (6849)	15% (9%–18%)	N = 10 (6849)	31% (16%–63%)	N = 7 (4577)	66% (51%–68%)	N = 3 (2,615)	16% (12%–26%)
Adult versus pediatric								
Adult	N = 41 (110,544)	21% (15%–39%)	N = 42 (106,315)	38% (23%–60%)	N = 25 (89,795)	66% (51%–70%)	N = 9 (8192)	16% (10%–36%)
Pediatric	N = 5 (4518)	20% (15%–21%)	N = 2 (355)	20% (17%–22%)	N = 5 (4518)	24% (17%–47%)	N = 0	NR

*AKI* acute kidney injury, *AKI*_*SC*_ AKI-positive according to SC criteria alone, *AKI*_*UO*_ AKI-positive according to UO criteria alone, *AKI*_*UOandSC*_ AKI-positive according to both UO and SC criteria—both tests collected, *AKI*_*UOorSC*_ AKI-positive according to UO criteria, SC criteria, or both—both tests collected, *CPB* cardiopulmonary bypass, *IQR* interquartile range, *N/A* not applicable, *NR* not reported

^a^Some studies contributed more than one data point for a given diagnostic method

^b^Stratified by mean age of population if reported

### Time to AKI diagnosis

**Fig. 4:**
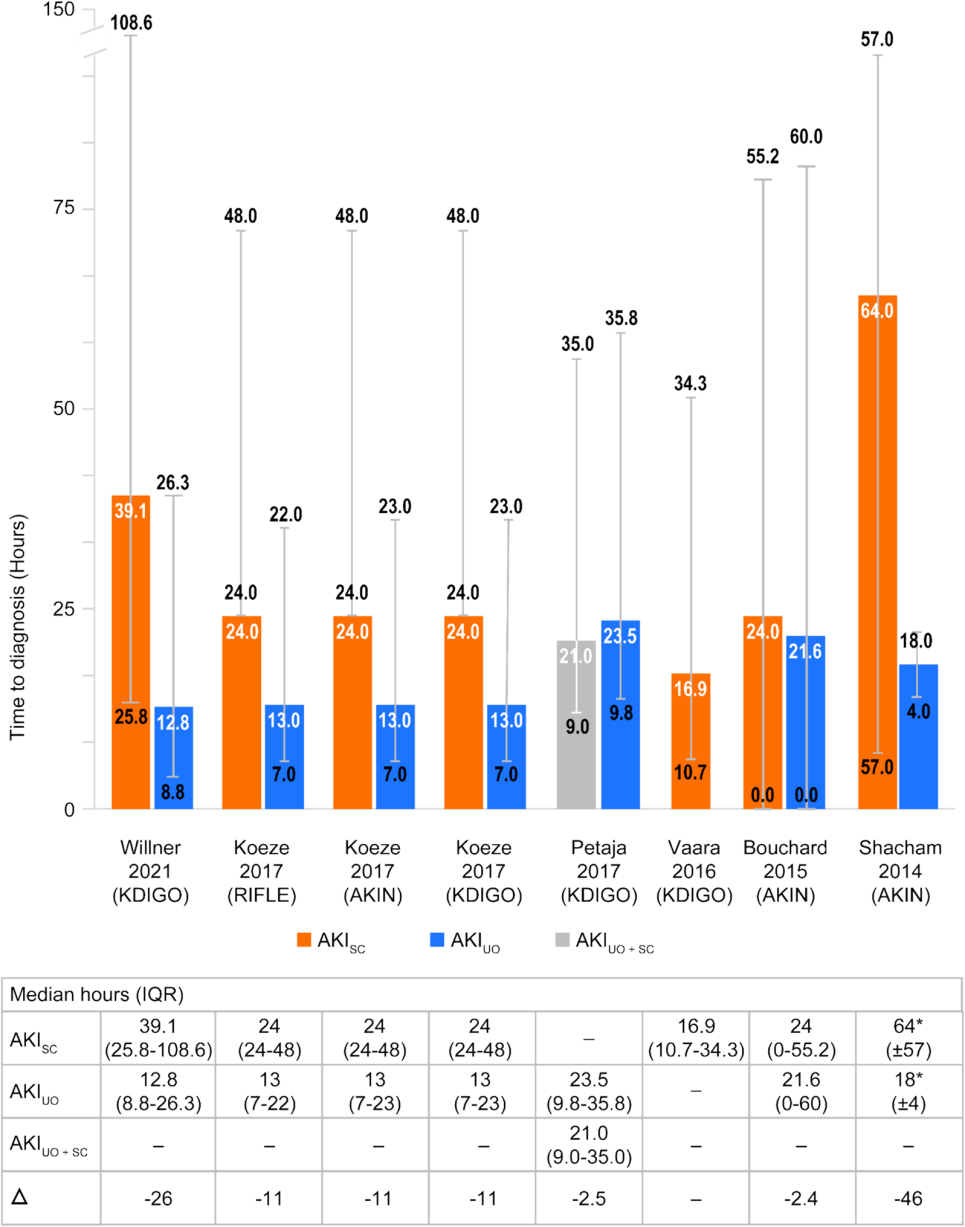
Time to AKI diagnosis – All patient populations. * Data presented as mean (± SD) where no median (IQR) was reported. Error bars correspond to the IQR or SD. *AKIN* Acute Kidney Injury Network, *AKI*_*SC*_ AKI-positive according to SC criteria alone, *AKI*_*UO*_ AKI-positive according to UO criteria alone, *AKI*_*UOandSC*_ AKI-positive according to both UO and SC criteria—both tests collected, *AKI*_*UOorSC*_ AKI-positive according to UO criteria, SC criteria, or both—both tests collected, *IQR* interquartile range, *KDIGO* Kidney Disease: Improving Global Outcomes, *RIFLE* Risk, Injury, Failure, Loss, and End-stage kidney disease, *SD* standard deviation, *VARC-2* Valve Academic Research Consortium-2

Specific reporting of timing of AKI diagnosis was identified in six studies (Fig. 4) [4, 24, 48, 59, 62, 75]. Overall, AKI was diagnosed 2.4 to 46.0 h sooner using UO criteria alone compared with SC criteria alone. In one study, AKI was diagnosed 2.5 h sooner using AKI_UO and SC_ criteria compared with SC criteria alone [59]. There was no information on time to AKI diagnosis based on AKI_UO or SC_ criteria. These findings remain consistent across all AKI classification criteria (i.e., KDIGO, RIFLE, AKIN, and VARC-2 criteria). Given the limited data on the timing of AKI diagnosis, subgroup analyses were not performed for this outcome.

### Adjusted mortality risk

**Fig. 5:**
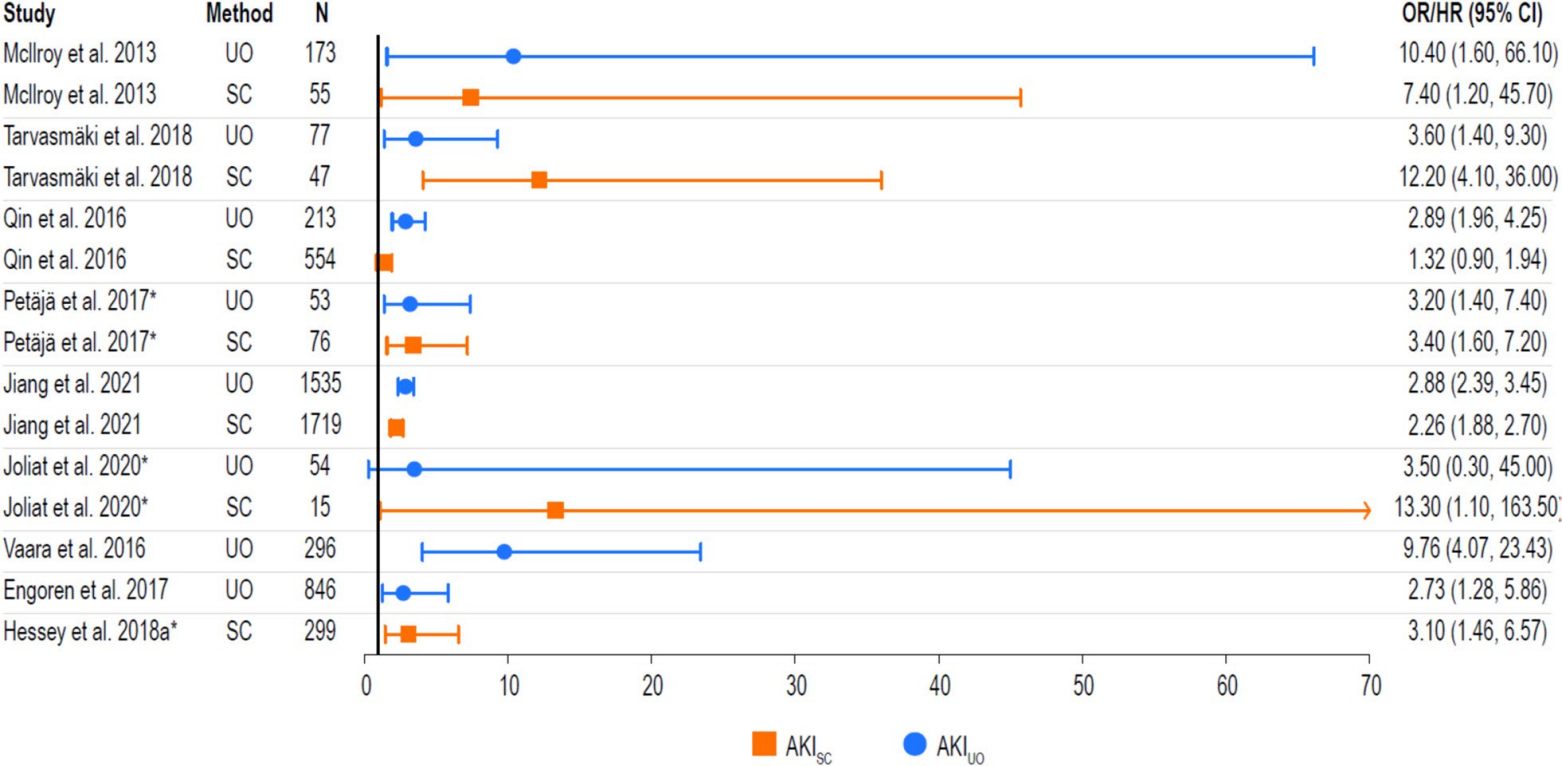
Adjusted mortality risk in AKI patients versus no AKI patients. *Data presented as HR (95% CI) where no OR (95% CI) was reported. Error bars correspond to the 95% CI and may extend past visible axis. **UO < 0.3 mL/kg/h for 6 h. The OR/HR of mortality is graphically represented per study and method as a point, with error bars as the 95%CI. The values for the OR/HR with the associated 95%CI are listed to the right of the figure. *AKI* acute kidney injury, *CI* confidence interval, *HR* hazard ratio, *OR* odds ratio, *SC* serum creatinine, *UO* urine output

Adjusted mortality risk in AKI patients (all stages) versus no AKI patients was reported in nine studies (Fig. 5) [45, 47, 50, 55, 58, 59, 61, 62]. These studies showed that UO is often a significant predictor of short- and long-term mortality in patients with AKI versus those without. Two studies also indicate that testing positive for both UO and SC is associated with the greatest mortality risk. In one study, the adjusted risk of mortality among patients diagnosed with AKI using UO or SC criteria (AKI_UO or SC_; HR: 3.38 [95% CI 1.63–7.02]) was comparable to those diagnosed with AKI using SC criteria alone (AKI_SC_; HR: 3.10 [95% CI 1.46–6.57]) [58].

In a 2021 report, Bianchi et al. found an association of 90-day mortality by UO criteria with stage 2 (OR: 2.43 [95% CI 1.57–3.77], p < 0.001) or stage 3 (OR: 6.24 [95% CI 3.69–10.52], p < 0.001) AKI after adjusting for several variables, including SC criteria and SC level [51]. For another study that focused on comparing more intensive (n = 2529) versus less intensive UO monitoring (n = 7461), for individuals diagnosed with AKI, results indicated that more intensive monitoring was associated with significantly reduced risk of 30-day mortality (HR: 0.90 [95% CI 0.81–0.99], p < 0.04) [20]. This same benefit was not seen for patients that received more intensive (n = 7973) versus less intensive (n = 2017) SC monitoring (HR: 1.10 [95% CI 0.98–1.24], p < 0.11). A detailed breakdown of adjusted mortality risk data (including by AKI stage) is summarized in Additional files 5 and 6.

### Length of stay

**Fig. 6:**
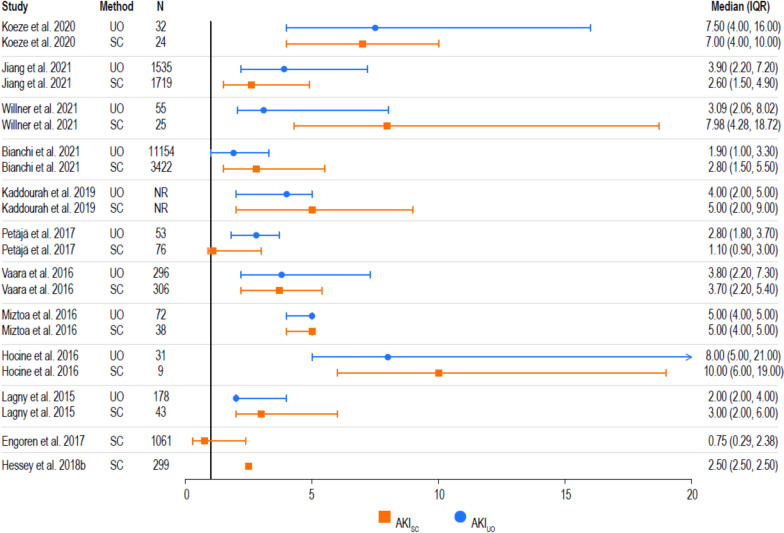
ICU length of stay among patients with AKI. The median ICU length of stay is graphically represented per study and method as a point, with error bars as the IQR. Median values with the associated IQR are listed to the right of the figure. Note that unadjusted results are presented. *AKI* acute kidney injury, *ICU* intensive care unit, *IQR* interquartile range, *SC* serum creatinine, *UO* urine output

Intensive care unit and hospital length of stay among patients with AKI were reported in 19 and 13 studies, respectively (Fig. 6). Overall, patients with AKI_UO_ had a median ICU length of stay that ranged from two to eight days and those with AKI_SC_ ranged from 0.75 to 10 days. The range was most broad for studies of patients diagnosed with combined criteria (i.e., 1.9 to 15 days with AKI_UO or SC_, and 2.8 to 15 days with AKI_UO and SC_; data not shown). These findings remain consistent among the studies that reported hospital length of stay in patients diagnosed with AKI (Additional file 7).

## Discussion

### General findings

Although UO is an important component of AKI detection, it is often omitted from clinical practice [17] as the recordings are often missed, late, or a challenge for nursing workflow [79]. Despite the present attempt to systematically review the impact of UO and SC on AKI-related outcomes across 50 clinical studies, clinically relevant characteristics and outcomes have been underreported. Based on available data, the incidence of AKI in hospitalized populations appeared to be highest when UO criteria were used in pooled populations and across each subgroup tested. Furthermore, the use of UO criteria allowed for AKI diagnosis earlier than SC criteria by 2.5 to 46 h. Similar to SC criteria, UO was found to be a strong predictor of AKI-related mortality.

### Acute kidney injury incidence

Interesting patterns of AKI detection were observed amongst subgroups. Except for the pediatric cohort, the incidence of AKI was greater with UO compared to SC criteria across several subgroups. This difference was especially pronounced in the cardiac patient subgroup, where the incidence of AKI measured by UO criteria was 2.5 times greater than SC criteria (45% versus 18%) and 3.2 times greater in those that underwent CPB (61% versus 19%). The increased detection of AKI by UO criteria is consistent with the implications of post-surgical fluid accumulation and imbalance, where SC may often be diluted [80, 81]. Fluid accumulation may lead to fluid accumulation syndrome reflecting in tissue edema, necessitating de-resuscitation active fluid removal by either diuretics or ultrafiltration) and close monitoring of UO [82]. A recent study of cardiac surgery patients with urinary catheter dwell times greater than 24 h found that all exhibited some degree of intra-abdominal hypertension, increasing the risk of AKI [83]. Increased AKI detection with UO criteria was also expected in older populations, where SC is reduced due to decreased muscle mass [84]. For patients over 60 years old, the greatest incidence was detected among AKI_UO or SC_ patients, indicating that more cases may be missed when omitting UO criteria from practice (66% compared to 20% by AKI_SC_). While UO measurements are robust in these populations, UO is still susceptible to artificial increases by therapies such as diuretics [85].

Recent studies have examined the detection of AKI within specific populations. In a study conducted by Zarbock et al., KDIGO Stage 1 AKI was detected more frequently by SC alone compared to UO alone (54% vs. 30%), whereas KDIGO stage 2 was detected more often by UO alone (40% vs. 28%) [86]. Zarbock et al. also observed that patients diagnosed by UO alone, regardless of KDIGO stage, had less persistent AKI. In a similar analysis, White et al. found that UO alone detected more sepsis-associated AKI than SC alone (44% vs. 35%); furthermore, patients diagnosed by UO alone had a three times greater chance of complete renal recovery [87]. Our findings and recent research highlight the importance of adhering to guidelines suggesting monitoring both UO and SC to diagnose AKI as early as possible for the greatest likelihood of detection. Additional studies are needed to further explore the prognostic value that may derive from examining one metric in the confirmed absence of the other.

### Impact of intensive urine output monitoring

Guidelines recommend that hourly monitoring of UO for a six-hour window and changes in SC within 48 h support timely definition of AKI [14]. In a large, retrospective study conducted by Jin et al. in 2017 of approximately 45,568 patients admitted to the ICU, intensive monitoring of UO (defined as hourly collection), compared with less intensive monitoring, resulted in increased detection of AKI and improved mortality outcomes for people with AKI, after adjustment for age and severity of illness [20]. These findings were supported by the present review, where AKI detection rates were higher in studies that collected UO measurements at an intensive frequency (maximum 1 h between measurements). However, it is unclear if these detection rates were a product of increased monitoring frequency, or if the populations included in the subgroup analyses were at higher risk for AKI. Hospitals that strive for hourly monitoring of UO are also likely attentive to the clinical and economic burden posed by infections associated with indwelling urinary catheters [88]—the very devices which facilitate efficient, accurate measurement of UO. Recently introduced, suction-assisted external wicking catheters can achieve the objective of reducing indwelling urinary catheter utilization while allowing for accurate urine output measurements without the necessity to weigh absorbent products or bed pads [89]. A study of urine capture in healthy volunteers found median urine capture rates for suction-assisted external wicking catheters exceeded 95% [90]. If hospitals verify these results in acute populations, suction-assisted external wicking catheters should prove to be effective alternatives for the early detection of AKI risk.

Across the eight studies that reported time-to-diagnosis outcomes, the use of AKI_UO_ criteria resulted in diagnosis 2.4 to 46.0 h earlier than AKI_SC_ criteria, which may support disease management efforts. As an example, hypovolemia is a primary factor in UO reductions, and is treated by initiation of fluid resuscitation [91]. Clinical observation has shown that renal function did not improve after fluid resuscitation in half of critically ill patients with oliguria [92]. As a result, oliguria unresponsive to fluid administration may be a strong predictive marker of AKI in hypovolemic patients. Similarly, UO monitoring may be important even after AKI diagnosis as UO is a therapeutic target representing a predictor of successful cessation during the 24 h prior to stopping continuous RRT [93]. Notably, patients are often kept on dialysis longer than may be appropriate, with a need for clinically- and cost-effective strategies that support successful and timely cessation of RRT [94]. Overall, there is a current lack of standardization in the implementation of AKI_UO_ criteria, and this may challenge clinical utility [38].

### Clinical and economic implications

The clinical and economic consequences of AKI can be substantial. For example, a 2017 study reporting on a 2012 National Inpatient Sample analysis of over 29 million patients demonstrated the excess costs associated with AKI in hospitalized patients. In this study, an incremental cost of $7933 and incremental length of stay of 3.2 days was reported in patients with AKI versus patients without AKI across variable diagnoses [95]. Such economic burden in the United States was noted to warrant further attention from hospitals and policymakers to enhance process of care. These data are further substantiated by a Canadian population-based study of 239,906 hospitalized patients which demonstrated that the severity of AKI, need for dialysis, and lack of kidney recovery were associated with significant healthcare costs persisting a year after admission. Furthermore, it was estimated that the incremental cost of AKI in Canada was estimated to be over $200 million dollars per year [96].

Recent developments in UO monitoring may be one step in helping to alleviate this economic burden. Urine output has been more recently established as a continuous, dynamic and low-cost parameter, providing improvements on manual collection methods, and allowing for real-time monitoring [19, 97, 98]. Notably, compared to manual UO monitoring, automated UO monitoring improves timeliness of documentation, and reduces hospital workloads without compromising accuracy [19]. As a result, it can be argued that continuous UO monitoring more closely adheres to AKI guidelines, and provides more data and information compared to a discrete laboratory parameter like SC [79]. One study demonstrated that a computerized decision support system that evaluates the patient for AKI every time a new UO value is charted in the clinical information system was found to reduce the progression of AKI from 42.0% of Stage 1 AKI patients to 33.5% of those Stage 1 patients [99]. Cost-effectiveness data on the impact of AKI detection automation is limited. A model of intensive UO monitoring resulting in early detection of AKI, and early initiation of renal replacement therapy in appropriate patients, estimated that the resultant shorter ICU length of stay would translate to organizational saving of $651 for each ICU patient [100]. Future studies should evaluate whether integration of automated and continuous UO measurements into the electronic health record allows for near-immediate implementation of AKI management strategies further improving upon these clinical results with decreased length of stay, hospital costs and mortality.

### Strengths and limitations of the present work

The current review represents a rigorous, objective, and systematic effort to comprehensively summarize the breadth of studies assessing outcomes with use of UO and/or SC criteria; no systematic reviews to date have reported diagnosis and outcomes data to this extent. Furthermore, the search included many newer studies that were published since the most recent guidelines (KDIGO, 2012 [14]). The present review was restricted to a qualitative analysis given the high heterogeneity of the included studies in design and patient characteristics. Key factors which may bias the detection of AKI were found to be heterogeneous (e.g., methods for establishing baseline SC, and use of hourly or average UO measurement) or not uniformly reported (e.g., history of chronic kidney disease, diuretic use, etc.). Variation in AKI incidence by up to 15% can result from the choice of methods used to estimate missing baseline serum creatinine levels [70]. Therefore, it is crucial to consider standardizing these methods. Furthermore, it is recognized that the monitoring of AKI is anticipated to be more frequent in high-risk populations, potentially leading to an inclination towards increased detection. This is evident in two studies that incorporated patients undergoing RRT, who might be at a more advanced stage of AKI disease progression, consequently potentially skewing the detection process [34, 35]. Although a lack of control for these factors is a notable limitation, the present study highlights the gaps in the current evidence base and the subsequent challenges associated with synthesizing the available information and making direct comparisons. Despite these challenges, subgroup analyses were conducted to identify unique trends and allow better interpretability of these data, where possible. Also, the design of the studies limited a true comparison of the longer-term impact of diagnosing with either SC or UO or a combination of criteria. For example, while many studies reported on the length of hospital or ICU stay, they were not designed to assess if earlier or more accurate diagnosis with one measure were significantly associated with a reduction in healthcare resource use, or disease progression. Additional research is needed to elucidate the mechanisms by which AKI_UO_ and AKI_SC_ lead to different outcomes, and this will likely vary by subgroup (for example, through influencing clinical decision-making). Ideally, future studies might be designed much like the study by Jin et al. from 2017 which appropriately controls for heterogeneity and potential confounding bias within the AKI-diagnosed cohort of patients. Finally, while the scope of this review focused on SC and UO as validated measures to diagnose AKI, additional work is needed to compare each to the evolving serum and urinary biomarkers as the field evolves [101, 102].

## Conclusions

Acute kidney injury is a multifactorial disease that results in significant healthcare resource utilization, contributing to prolonged lengths of ICU and hospital stay, and increased mortality [2–8, 10, 95, 103]. While diagnostic criteria for AKI have been standardized, variable hospital compliance for UO components of guidelines impedes researchers from assessing the total potential impact of AKI care guidelines on patient outcomes [11]. Despite technical difficulties associated with accurate intermittent manual measurement [17], UO in combination with SC is the most sensitive indicator of AKI with UO alone offering additional prognostic value compared to SC alone. Further, UO monitoring appears to identify AKI earlier than SC monitoring alone offering opportunity for earlier intervention. Future data synthesis that aligns with these findings are contemplated in the Scope of Work for the KDIGO AKI/Acute Kidney Disease Guideline Update, which will examine whether and how UO should be combined with SC criteria for defining and staging AKI [104]. The Scope of Work will also examine whether UO should be weighted differently from SC criteria, and how weighting may vary for each stage of AKI.

### Supplementary Information

Additional file 1.

Additional file 2.

Additional file 3.

Additional file 4.

Additional file 5.

Additional file 6.

Additional file 7.

## Data Availability

All data generated or analyzed during this study are included in this published article [and its additional information files].

## Abbreviations

AKI: Acute kidney injury
AKI_SC_: AKI-positive according to SC criteria alone
AKI_UO_: AKI-positive according to UO criteria alone
AKI_UO and SC_: AKI-positive according to both UO and SC criteria—both tests collected
AKI_UO or SC_: AKI-positive according to UO criteria, SC criteria, or both—both tests collected
AKIN: Acute Kidney Injury Network
CAN: Canadian dollars
CPB: Cardiopulmonary bypass
CENTRAL: Cochrane central register of controlled trials
CI: Confidence interval
CKD: Chronic kidney disease
COVID-19: Coronavirus disease 2019
HR: Hazard ratio
ICU: Intensive care unit
IQR: Interquartile range
KDIGO: Kidney Disease: Improving Global Outcomes
OR: Odds ratio
PICOS: Population, intervention, comparator, outcomes, and study design
PRESS: Peer review of electronic search strategies
PRISMA: Preferred reporting items for systematic reviews and meta-analyses
NICE: National institute for health and care excellence
NOS: Newcastle–ottawa quality assessment scale
RIFLE: Risk, injury, failure, loss of kidney function, and End-stage kidney disease
RCT: Randomized controlled trial
RRT: Renal replacement therapy
SAPS: Simplified Acute Physiology Score
SC: Serum creatinine
SLR: Systematic literature review
UO: Urine output
USD: United States Dollar
VARC-2: Valve Academic Research Consortium-2
