# Supplementary material for: Urine output is an early and strong predictor of acute kidney injury and associated mortality: a systematic literature review of 50 clinical studies
Source: Ann Intensive Care. 2024 Jul 9;14:110. doi: 10.1186/s13613-024-01342-x (PMC11233478; doi:10.1186/s13613-024-01342-x)
Supplement: Supplementary file 1 — Additional file 1. [file 13613_2024_1342_MOESM1_ESM.docx]

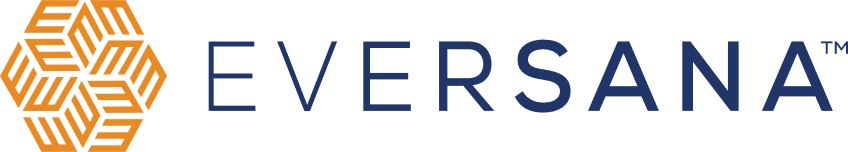


Urine Output as a Measure of Acute Kidney Injury

Protocol for Systematic Literature Review

| **Prepared For:** | Tim Kelly |
| --- | --- |
| **Company:** | BD |
| **Date:** | January 26, 2023 |

204-3228 South Service Road, Burlington, ON L7N 3H8 Canada
T: 905.637.6231 | F: 905.637.5014 | www.eversana.com

Table of Contents

[Administrative Structure 2](#_Toc113615803)

[1.0 Introduction 3](#_Toc113615804)

[2.0 Methods 3](#_Toc113615805)

[2.1 Protocol 3](#_Toc113615806)

[2.2 Search Strategy 3](#_Toc113615807)

[2.3 Eligibility Criteria 3](#_Toc113615808)

[2.4 Data Extraction 5](#_Toc113615809)

[2.5 Quality Assessment 5](#_Toc113615810)

[2.6 Reporting 5](#_Toc113615811)

[References 7](#_Toc113615812)

[Appendix A PRISMA-P Checklist 8](#_Toc113615813)

[Appendix B Search Strategy 10](#_Toc113615814)

[Appendix C Press Checklist 15](#_Toc113615815)

# Administrative Structure

Sponsor: BD

Tim Kelly, Tim.Kelly@bd.com

Sponsors were involved in the creation and development of this protocol.

Operator: EVERSANA

**Project Lead:** Nicole Ferko (Vice President, Value & Evidence Services), nicole.ferko@eversana.com

**Project Team:** Evelyn Worthington (Associate Director), evelyn.worthington@eversana.com

Krista Tantakoun (Research Assistant II), Anthony Zara (Intern), Joanna Bielecki (Information Specialist)

EVERSANA

Value & Evidence Division

Suite 204, 3228 South Service Road

Burlington, Ontario, L7N 3H8 Canada

Researcher Prof Dr Manu Malbrain

First Department of Anaesthesiology and Intensive Therapy

Medical University Lublin, Poland

# Introduction

***Becton, Dickinson and Company (BD)*** (***the Client***) is an industry leader in drug delivery systems, especially in chronic disease areas. Their varied product offering includes needle technologies, pre-fillable syringes, safety/shielding systems, and self-injection (i.e., on-body devices) systems. The target audience for these products include pharmaceutical and biotech companies across the globe.

***The Client*** has requested that EVERSANA perform a systematic literature review (SLR) on urinary output (UO) as an indicator for acute kidney injury (AKI) with special consideration for comparisons between UO and serum creatinine (SC). The intention of the SLR is to complement other ongoing research and to provide insight that perhaps an overreliance on SC values as a trigger for AKI means that they are diagnosing the condition later, and potentially less of it, than if UO were the trigger, especially in critically ill patients with fluid accumulation and hemodilution when SC values tend to be lower.

# Methods

## Protocol

This protocol was developed between ***The Client*** and ***EVERSANA*** and outlines the PICOS (Population, Intervention, Comparators, Outcomes, and Study design) criteria and methodology for the SLR. This protocol followed the Preferred Reporting Items for Systematic Reviews and Meat-Analyses Protocols (PRISMA-P) guidelines, and a PRISMA-P checklist can be found in [**Appendix A**](#_PRISMA-P_Checklist).

## Search Strategy

The search strategy was developed based on pre-defined PICOS criteria through an iterative process by an experienced medical information specialist in consultation with the review team (see **Appendix B**). Published literature will be identified for the SLR by searching MEDLINE^®^, MEDLINE^®^ In-Process, and Other Non-Indexed Citations, Embase, and the Cochrane Central Register of Controlled Trials using a combination of controlled vocabulary and keywords relevant to the research question (see a detailed PICOS list in **Table 1**). Prior to execution, the MEDLINE^®^ search was previously peer reviewed by an independent information specialist using the Peer Review of Electronic Search Strategies[^1^](#_ENREF_1) (PRESS) Checklist (see **Appendix C**). After the MEDLINE^®^ strategy was finalized and peer-reviewed, it was adapted to the syntax and subject headings of the other databases.

The search strategy was designed to identify relevant studies on UO as an indicator of AKI, including comparative studies between UO and SC published within the last ten years in the English language. Single-arm studies, conference abstracts, posters, and narrative reviews will be excluded. On-topic published SLRs and meta-analyses will be formally excluded; however, records will be retained, and particularly relevant articles will be leveraged to cross-reference the bibliography of the SLR for completeness, as well as to inform background information and project direction.

Table 1: Detailed Criteria for Inclusion and Exclusion of Studies

| **Criteria** | **Include** | **Exclude** |
| --- | --- | --- |
| **Population** | - Patients assessed for AKI in acute care settings - Adults - Pediatrics | - Patients not assessed for AKI - Infants, pre-term infants - Newborns - Studies with fewer than 20 patients - Patients who are pregnant |
| **Intervention** | - UO as a diagnostic for AKI - Combination of SC and UO | - Other diagnostic biomarkers ^a^ |
| **Comparator** | - SC as a diagnostic for AKI | - Other diagnostic biomarkers ^a^ |
| **Outcomes** | - Accuracy of detection, prediction, and timing of AKI diagnosis - AKI progression, movement to more advanced stages of AKI, movement to ARF, CKD, or ESRD - Morbidity: increase/decrease in SOFA, fluid accumulation, (biomarkers for) fluid accumulation syndrome - Mortality (or other survival outcomes) related to diagnosis of AKI - Healthcare resource utilization related to diagnosis of AKI, such as: - ICU discharge / *exitus* status or ward transfers - Organ failure free days, vasopressor free days, ventilator free days, etc. - Hospitalizations, emergency room visits, and outpatient physician visits - ICU or hospital length of stay - The use of renal replacement therapy or other bedside hemodialysis therapy - The use of diuretics to treat AKI | - Prevalence of AKI without a comparator of UO with SC as the identification criteria - Long-term outcomes of AKI, ARF, CKD, ESRD without a focus on diagnosis or AKI stage progression |
| **Study Design** | - Comparative studies: - Clinical studies - Non-randomized controlled trials - Randomized controlled trials - Observational studies | - Non-comparative studies - Case studies - Opinion pieces - Letters - Editorials - Narrative reviews - Conference abstracts and posters - Off-topic SLRs and meta-analyses - On-topic SLRs and meta-analyses will be formally excluded^b^ at the title-and-abstract stage of screening, but the most relevant record(s) will be cross-referenced with results of the SLR |
| **Location** | Global | None |
| **Language** | English only | Non-English |
| **Date of Publication** | The past ten years (i.e., 2012 or later) | Published more than 10 years ago (i.e., 2011 or earlier) |

^a^ These studies will be included if they employed both UO and SC in the evaluation of the biomarker. Studies comparing biomarkers will be included during title-and-abstract screening if (1) otherwise relevant and (2) the biomarker is compared to UO or SC as a diagnostic for AKI (i.e., the study presents data related to the accuracy of detection, prediction, and timing of AKI related to one of UO or SC). These studies will then be excluded during full-text screening to facilitate retrieval in the event of future needs.

^b^ These studies will be included during title-and-abstract screening if otherwise relevant, and then excluded during full-text screening. The most recent and relevant SLRs/meta-analyses may be cross-referenced against the results of this SLR.

Abbreviations: AKI = acute kidney injury; ARF = acute renal failure; CKD = chronic kidney disease; ESRD = end-stage renal disease; ICU = intensive care unit; SC = serum creatinine; SLR = systematic literature review; SOFA = Sequential Organ Failure Assessment; UO = urine output

## Eligibility Criteria

Two reviewers will review the study records, citation titles, and abstracts identified in the literature search to assess study eligibility based on PICOS criteria. Potentially eligible citations will be reviewed in full-text form for formal inclusion in the final review. An independent reviewer not involved in the process will assess eligibility if there are disagreements among the two reviewers. During full-text review, the reviewers will also document reasons for exclusion and present the results in the form of a PRISMA flow diagram.

Studies that evaluate patients for AKI in the acute care setting will be eligible for inclusion. Studies with a very small population (i.e., fewer than 20 patients), or that only include infants or newborns, will be excluded.

Studies that evaluate SC and UO as a diagnostic indicator of AKI will be eligible for inclusion. The AKI criteria according to different guidelines are summarized in **Table 2**. Studies that evaluate only SC or UO (i.e., are not comparative) and are not specific to AKI diagnosis or stage progression will be excluded. Additionally, studies comparing out-of-scope biomarkers (e.g., creatinine clearance, neutrophil gelatinase-associated lipocalin [NGAL], cystatin C, urinary tissue inhibitor of metalloproteinases-2 (TIMP-2), and insulin-like growth factor binding protein 7 (IGFBP7), etc.) to one of UO or SC for the diagnosis of AKI will be included at the title-and-abstract stage of screening if (1) otherwise relevant and (2) the biomarker is compared to UO or SC as a diagnostic for AKI (i.e., the study presents data related to the accuracy of detection, prediction, and timing of AKI related to one of UO or SC). These will then be excluded at full-text stage of screening to facilitate retrieval in the event of future needs.

Table 2. AKI criteria according to different guidelines

| **Stage** | **RIFLE** | **AKIN** | **KDIGO** |
| --- | --- | --- | --- |
| **Stage 1/ Risk** | SC 1.5x baseline (within 7 days)  Or  GFR decrease >25%  Urine Output <0.5ml/kg/h x 6h | SC 1.5-2.0 x baseline (within 7 days)  Or  ⋝0.3mg/dl increase (within 48h)  Urine Output <0.5ml/kg/h x 6h | SC 1.5-1.9 x baseline (within 7 days)  Or  ⋝0.3mg/dl increase (within 48h)  Urine Output <0.5ml/kg/h x 6h |
| **Stage 2/ Injury** | SC 2 x baseline  Or  GFR decrease >50%  Urine Output <0.5ml/kg/h x 12h | SC 2-3 x baseline  Urine Output <0.5ml/kg/h x 12h | SC 2-2.9 x baseline  Urine Output <0.5ml/kg/h x 12h |
| **Stage 3/ Failure** | SC 3 x baseline  Or  GFR decrease >75%  Or  SC⋝4 (with acute rise ⋝ 0.5mg/dl)  Urine Output <0.3ml/kg/h x 24h  Or  Anuria x 12h | SC 3 x baseline  Or  SC⋝4 (with acute rise ⋝ 0.5mg/dl)  Or  Initiation of kidney replacement therapy  Urine Output <0.3ml/kg/h x 24h  Or  Anuria x 12h | SC 3 x baseline  Or  SC⋝4 (with ⋝ 0.3mg/dl increase within 48h or 1.5 x baseline)  Or  Initiation of kidney replacement therapy  Urine Output <0.3ml/kg/h x 24h  Or  Anuria x 12h |
| **Loss of kidney function** | Complete loss of kidney function > 4 weeks |  |  |
| **End-stage kidney disease** | End-stage kidney disease (> 3 months) |  |  |

Abbreviations: AKIN = acute kidney injury network; KDIGO = Kidney Disease: Improving Global Outcomes; RIFLE = Risk, Injury, Failure, Loss of kidney function, End-stage kidney disease; SC = serum creatinine

Studies evaluating the accuracy (further defined as timeliness and comprehensiveness) of detection and prediction, mortality (or other survival outcomes), or healthcare resource utilization related to AKI diagnosis will be eligible for inclusion. Studies that only evaluate the comparative incidence or prevalence of AKI, or that only assess long-term outcomes of AKI without a focus on diagnosis or AKI stage progression, will be excluded. Studies evaluating the association between perioperative or intraoperative measurements of SC or UO and the incidence of post-operative AKI will also be excluded. These studies will be tagged to facilitate retrieval in the event of future needs.

## Data Extraction

Using a standardized Excel worksheet, relevant details to reflect the previously outlined categories will be collected. For example, general study information, study characteristics (e.g., study design, setting, inclusion/exclusion criteria, intervention protocols, etc.), population and baseline characteristics (e.g., age, gender, baseline treatment experience, mechanism of diagnosing AKI), results (e.g., time to diagnosis of AKI, incidence of AKI), and data sources will be collected. The KDIGO care bundle elements will also be checked for (**Table 3**). An independent reviewer not involved in the data collection process will review data extraction and document quality review throughout. An independent reviewer not involved in the data collection process would resolve any discrepancies between reviewers.

Table 3: KDIGO Care Bundle Elements

| **Bundle element** | **Criteria for compliance** |
| --- | --- |
| Discontinuation of all nephrotoxic agents | Patients did not receive any nephrotoxic substances (e.g., NSAIDs, vancomycin, aminoglycosides) |
| Optimization of hemodynamics | Patient’s lowest documented MAP was >65mmHg and no treatment had been initiated or optimized to achieve this goal |
| Close monitoring of serum creatinine, urine output and fluid balance | Serum creatinine was measured twice a day, and  urine output was documented at least every 2 hours, and fluid balance was recorded twice a day |
| Avoidance of hyperglycemia | Blood glucose was not ≥150mg/dl on two consecutive samples more than 3 hours apart |
| Consideration of alternatives to radiocontrast agents | Patients did not receive radiocontrast agents for the first 72 hours after surgery |
| Discontinuation of ACEi/ARBs | Patients did not receive ACEi/ARBs during the first 48h after surgery |
| Avoidance of HES, gelatin, and chloride-rich solutions | Patients did not receive HES, gelatin or chloride rich solutions for 72h after surgery |

Abbreviations: ACEi = angiotensin-converting-enzyme inhibitors; ARBs = angiotensin receptor blockers; HES = hydroxyethyl starch; KDIGO = Kidney Disease: Improving Global Outcomes; MAP = mean arterial pressure; NSAIDs = nonsteroidal anti-inflammatory drugs

## Quality Assessment

Supplier will assess the quality of the included studies available in full-text only using appropriate quality assessment tools that are recommended by major health technology assessment (HTA) agencies such as the National Institute of Health and Excellence (NICE) and the Canadian Agency for Drugs and Technologies in Health (CADTH), for example, the NICE single technology appraisal (STA) quality checklist. Comparative studies will be assessed. Optionally, single-arm studies could also be assessed for additional budget (e.g., methodological index for non-randomized studies [MINORS] and Newcastle-Ottawa Scale [NOS] for cohort and case-control studies).

## Reporting

***EVERSANA*** will prepare a slide deck summarizing the methods and results of the SLR. The final slide deck will include an executive summary, introduction, objectives, methods, results, and discussion. The slide deck will focus primarily on results and conclusions including “key takeaways” and “gaps”.

***EVERSANA*** will also prepare a manuscript summarizing the methods and results of the SLR and/or methods and results of SLR-related additional activities (e.g., feasibility assessments or meta-analyses). Manuscript preparation will be conducted in liaison with ***The Client*** and outside clinical advisors (i.e., key opinion leaders [KOLs]) in terms of content, direction, manuscript outline, and manuscript drafts.

A technical report is not planned for this project.

# References

^1^McGowan J, Sampson M, Salzwedel DM, Cogo E, Foerster V et al. (2016) PRESS peer review of electronic search strategies: 2015 guideline statement. *Journal of clinical epidemiology* 75 40-46.

1. PRISMA-P Checklist

**PRISMA-P (Preferred Reporting Items for Systematic review and Meta-Analysis Protocols) 2015 checklist: recommended items to address in a systematic review protocol***

| **Section and topic** | **Item No** | **Checklist item** | **Section Number** |
| --- | --- | --- | --- |
| **ADMINISTRATIVE INFORMATION** | | |  |
| **Title:** | | | |
| Identification | 1a | Identify the report as a protocol of a systematic review | Title page and Section 1 |
| Update | 1b | If the protocol is for an update of a previous systematic review, identify as such | NA |
| Registration | 2 | If registered, provide the name of the registry (such as PROSPERO) and searregistration number | NA |
| **Authors**: | | | |
| Contact | 3a | Provide name, institutional affiliation, e-mail address of all protocol authors; provide physical mailing address of corresponding author | Administrative Structure |
| Contributions | 3b | Describe contributions of protocol authors and identify the guarantor of the review | NA |
| Amendments | 4 | If the protocol represents an amendment of a previously completed or published protocol, identify as such and list changes; otherwise, state plan for documenting important protocol amendments | NA |
| **Support**: | | | |
| Sources | 5a | Indicate sources of financial or other support for the review | Administrative Structure |
| Sponsor | 5b | Provide name for the review funder and/or sponsor | Administrative Structure |
| Role of sponsor or funder | 5c | Describe roles of funder(s), sponsor(s), and/or institution(s), if any, in developing the protocol | Administrative Structure |
| **INTRODUCTION** | | |  |
| Rationale | 6 | Describe the rationale for the review in the context of what is already known | Section 1.0 |
| Objectives | 7 | Provide an explicit statement of the question(s) the review will address with reference to participants, interventions, comparators, and outcomes (PICO) | Sections 1.0, 2.0 |
| **METHODS** | | |  |
| Eligibility criteria | 8 | Specify the study characteristics (such as PICO, study design, setting, time frame) and report characteristics (such as years considered, language, publication status) to be used as criteria for eligibility for the review | Section 2.3 |
| Information sources | 9 | Describe all intended information sources (such as electronic databases, contact with study authors, trial registers or other gray literature sources) with planned dates of coverage | Section 2.2 |
| Search strategy | 10 | Present draft of search strategy to be used for at least one electronic database, including planned limits, such that it could be repeated | Appendix B |
| **Study records**: |  |  |  |
| Data management | 11a | Describe the mechanism(s) that will be used to manage records and data throughout the review | Section 2.4 |
| Selection process | 11b | State the process that will be used for selecting studies (such as two independent reviewers) through each phase of the review (that is, screening, eligibility and inclusion in meta-analysis) | Section 2.3 |
| Data collection process | 11c | Describe planned method of extracting data from reports (such as piloting forms, done independently, in duplicate), any processes for obtaining and confirming data from investigators | Section 2.4 |
| Data items | 12 | List and define all variables for which data will be sought (such as PICO items, funding sources), any pre-planned data assumptions and simplifications | Section 2.3 |
| Outcomes and prioritization | 13 | List and define all outcomes for which data will be sought, including prioritization of main and additional outcomes, with rationale | Section 2.3 |
| Risk of bias in individual studies | 14 | Describe anticipated methods for assessing risk of bias of individual studies, including whether this will be done at the outcome or study level, or both; state how this information will be used in data synthesis | Section 2.5 |
| Data synthesis | 15a | Describe criteria under which study data will be quantitatively synthesised | NA |
|  | 15b | If data are appropriate for quantitative synthesis, describe planned summary measures, methods of handling data and methods of combining data from studies, including any planned exploration of consistency (such as I^2^, Kendall’s τ) | NA |
|  | 15c | Describe any proposed additional analyses (such as sensitivity or subgroup analyses, meta-regression) | NA |
|  | 15d | If quantitative synthesis is not appropriate, describe the type of summary planned | NA |
| Meta-bias(es) | 16 | Specify any planned assessment of meta-bias(es) (such as publication bias across studies, selective reporting within studies) | NA |
| Confidence in cumulative evidence | 17 | Describe how the strength of the body of evidence will be assessed (such as GRADE) | NA |

*** It is strongly recommended that this checklist be read in conjunction with the PRISMA-P Explanation and Elaboration (cite when available) for important clarification on the items. Amendments to a review protocol should be tracked and dated. The copyright for PRISMA-P (including checklist) is held by the PRISMA-P Group and is distributed under a Creative Commons Attribution Licence 4.0.**

Source: Shamseer L, Moher D, Clarke M, Ghersi D, Liberati A, Petticrew M, Shekelle P, Stewart L, PRISMA-P Group. Preferred reporting items for systematic review and meta-analysis protocols (PRISMA-P) 2015: elaboration and explanation. BMJ. 2015 Jan 2;349(jan02 1):g7647.

1. Search Strategy

Ovid Multifile

Database: EBM Reviews - Cochrane Central Register of Controlled Trials <June 2022>, EBM Reviews - Cochrane Database of Systematic Reviews <2005 to June 29, 2022>, Embase <1974 to 2022 July 05>, Ovid MEDLINE(R) and Epub Ahead of Print, In-Process, In-Data-Review & Other Non-Indexed Citations and Daily <1946 to July 05, 2022>

Search Strategy:

--------------------------------------------------------------------------------

1 Acute Kidney Injury/ or (acute$ adj3 (renal$ or kidney?) adj3 (injur$ or fail$ or insufficien$ or nonsufficien$ or non-sufficien$ or trauma? or harm? or damage?)).tw,kf,kw. [Acute Kidney Injury Terms] (193551)

2 ((Urodynamics/ or Urine/ or Urine Specimen Collection/ or Acute Kidney Injury/ur) and (output? or volume? or amount? or measure$ or quantity or quantities).tw,kf,kw.) or ((urin$ adj2 (output? or volume? or amount? or measure$ or quantity or quantities)) or ((postvoid$ or post-void$ or void$) adj3 residual) or urodynamic$).tw,kf,kw. [Urine Output Terms] (162844)

3 Creatinine/bl, ur or (creatinine$ adj3 (serum or sera or blood$ or plasma?)).tw,kf,kw. [Serum Creatinine Terms] (202168)

4 1 and 2 and 3 [AKI and UO and SC ] (3827)

5 1 and 2 [AKI and UO] (8053)

6 1 and 3 [AKI and SC] (29748)

7 exp Animals/ not (exp Animals/ and Humans/) [ANIMAL STUDIES ONLY - REMOVE - MEDLINE] (17089654)

8 (address or autobiography or bibliography or biography or comment or dictionary or directory or editorial or "expression of concern" or festschrift or historical article or interactive tutorial or lecture or legal case or legislation or news or newspaper article or patient education handout or personal narrative or portrait or video-audio media or webcast or (letter not (letter and randomized controlled trial))).pt. [Opinion publications - Remove -MEDLINE] (4688108)

9 4 not (7 or 8) [AKI and UO and SC - Animals and Opinion Publications - removed] (2764)

10 5 not (7 or 8) [AKI and UO - Animals and Opinion Publications - removed] (5601)

11 6 not (7 or 8) [AKI and SC - Animals and Opinion Publications - removed] (19533)

12 (randomized controlled trial or controlled clinical trial).pt. or (randomized or placebo or randomly or trial or groups).ab. or drug therapy.fs. [RCTs – MEDLINE sensitive Filter – Cochrane HSSS, 2019] (14730710)

13 exp Randomized Controlled Trials as Topic/ or Clinical Trial, Phase II/ or Clinical Trial, Phase III/ or (equivalence trial or pragmatic clinical trial).pt. or (randomised or randomi#ation? or RCT or placebo$ or ((singl$ or doubl$ or trebl$ or tripl$) adj (mask$ or blind$ or dumm$)) or ((study or trial or CT) adj3 (phase 2 or phase 2a or phase 2b or phase 2c or phase II or phase IIa or phase IIb or phase IIc or phase 3 or phase 3a or phase 3b or phase 3c or phase III or phase IIIa or phase IIIb or phase IIIc or "phase? 2/3" or "phase? II/III")) or open label$).tw,kw,kf. [PHASE 2-3, OPEN LABEL - ADDITIONAL TERMS TO SUPPLEMENT RCTs FILTER] (2368347)

14 Cohort studies/ or comparative study/ or follow-up studies/ or prospective studies/ or risk factors/ or cohort.mp. or compared.mp. or groups.mp. or multivariate.mp. [NON-RANDOMIZED STUDIES – MEDLINE Filter - sensitive, Furlan,2006] (20119353)

15 Comparative study/ or Follow-up studies/ or Time factors/ or (preoperat$ or pre operat$).mp. or (chang$ or evaluat$ or reviewed or prospective$ or retrospective$ or baseline or cohort or case series).tw. [OBSERVATIONAL STUDIES – MEDLINE Filter – max specificity, Fraser, 2006] (25021958)

16 non-randomized controlled trials as topic/ or controlled before-after studies/ or interrupted time series analysis/ or historically controlled study/ or case-control studies/ or cross-sectional studies/ or observational study/ or (((cohort or concurrent or non-concurrent or incidence or follow-up or followup or longitudinal or prospective or retrospective or nonrandom$ or non-random$ or quasi-random$ or quasi-experiment$ or quasirandom$ or quasiexperiment$ or pretest or posttest or pre-test or post-test or "before after" or CBA or ITS or (historical$ adj2 control$) or case-control$ or case-comparison or case-compeer or case-referrent or case-referent or case-base or cross-section$ or prevalence) adj3 (stud$ or design?)) or non-RCT or nRCT or real-world or RWE or regist$ or (interrupted adj2 time adj2 series)).tw,kw,kf. [ADDITIONAL TERMS TO SUPPLEMENT NRS FILTERS] (6321847)

17 12 or 13 or 14 or 15 or 16 [All Study Types (except single-arm)] (36952968)

18 9 and 17 [AKI and UO and SC All Study Types (except single-arm)] (2447)

19 (("single arm" or noncomparativ* or non-comparativ* or noncomparison* or non-comparison*) adj3 (cohort? or study or studies or trial?)).tw,kw,kf. [SINGLE-ARM STUDIES – MEDLINE] (33830)

20 10 and 19 [AKI and UO and Single-Arm Studies Only] (8)

21 11 and 19 [AKI and SC and Single-Arm Studies Only] (20)

22 18 or 20 or 21 (2465)

23 22 use ppez [MEDLINE RECORDS] (1065)

24 acute kidney failure/ or (acute$ adj3 (renal$ or kidney?) adj3 (injur$ or fail$ or insufficien$ or nonsufficien$ or non-sufficien$ or trauma? or harm? or damage?)).tw,kf,kw. [Acute Kidney Injury Terms] (214500)

25 urine sampling/ or ((urodynamics/ or urine/) and (output? or volume? or amount? or measure$ or quantity or quantities).tw,kf,kw.) or ((urin$ adj2 (output? or volume? or amount? or measure$ or quantity or quantities)) or ((postvoid$ or post-void$ or void$) adj3 residual) or urodynamic$).tw,kf,kw. [Urine Output Terms] (174332)

26 creatinine blood level/ or (creatinine$ adj3 (serum or sera or blood$ or plasma?)).tw,kf,kw. (236548)

27 24 and 25 and 26 [AKI and UO and SC ] (3937)

28 24 and 25 [AKI and UO] (8109)

29 24 and 26 [AKI and SC] (37015)

30 (exp animal/ or exp animal experimentation/ or exp animal model/ or exp animal experiment/ or nonhuman/ or exp vertebrate/) not (exp human/ or exp human experimentation/ or exp human experiment/) [ANIMAL STUDIES ONLY - REMOVE - EMBASE] (11978959)

31 (editorial or letter).pt. not randomized controlled trial/ [OPINION PIECES REMOVE - Embase] (3751379)

32 27 not (30 or 31) [AKI and UO and SC - Animals and Opinion Publications - removed] (3588)

33 28 not (30 or 31) [AKI and UO - Animals and Opinion Publications - removed] (7215)

34 29 not (30 or 31) [AKI and SC - Animals and Opinion Publications - removed] (30282)

35 Randomized controlled trial/ or Controlled clinical study/ or randomization/ or intermethod comparison/ or double blind procedure/ or human experiment/ or (compare or compared or comparison or trial).ti. or ((evaluated or evaluate or evaluating or assessed or assess) and (compare or compared or comparing or comparison)).ab. or (random$ or placebo or (open adj label) or ((double or single or doubly or singly) adj (blind or blinded or blindly)) or parallel group$1 or (crossover or cross over) or ((assign$ or match or matched or allocation) adj5 (alternate or group$1 or intervention$1 or patient$1 or subject$1 or participant$1)) or (assigned or allocated) or (controlled adj7 (study or design or trial)) or (volunteer or volunteers)).ti,ab. (10964624)

36 (Cross-sectional study/ not (randomized controlled trial/ or controlled clinical study/ or controlled study/ or randomi?ed controlled.ti,ab. or control group$1.ti,ab.)) or ((((case adj control$) and random$) not randomi?ed controlled) or (nonrandom$ not random$) or "Random field$" or (random cluster adj3 sampl$)).ti,ab. or (Systematic review not (trial or study)).ti. or ((review.ab. and review.pt.) not trial.ti.) or ("we searched".ab. and (review.ti. or review.pt.)) or ("update review" or (databases adj4 searched)).ab. or ((rat or rats or mouse or mice or swine or porcine or murine or sheep or lambs or pigs or piglets or rabbit or rabbits or cat or cats or dog or dogs or cattle or bovine or monkey or monkeys or trout or marmoset$1).ti. and animal experiment/) or (Animal experiment/ not (human experiment/ or human/)) (5658334)

37 35 not 36 [RCTs – Embase sensitive Filter – Cochrane HSSS, 2019] (10001494)

38 phase 2 clinical trial/ or phase 3 clinical trial/ or (equivalence trial or pragmatic clinical trial).pt. or (randomised or randomi#ation? or RCT or placebo* or ((singl* or doubl* or trebl* or tripl*) adj (mask* or blind* or dumm*)) or ((study or trial or CT) adj3 (phase 2 or phase 2a or phase 2b or phase 2c or phase II or phase IIa or phase IIb or phase IIc or phase 3 or phase 3a or phase 3b or phase 3c or phase III or phase IIIa or phase IIIb or phase IIIc or "phase? 2/3" or "phase? II/III")) or open label*).tw,kw,kf. [PHASE 2-3, OPEN LABEL - ADDITIONAL TERMS TO SUPPLEMENT RCTs FILTER] (2109929)

39 Clinical article/ or controlled study/ or major clinical study/ or prospective study/ or cohort.mp. or compared.mp. or groups.mp. or multivariate.mp. [NON-RANDOMIZED STUDIES– Embase Filter - sensitive, Furlan,2006] (24170198)

40 Controlled study/ or Treatment outcome/ or Major clinical study/ or Clinical trial/ or (chang$ or evaluat$ or reviewed or baseline or (compare$ or compara$)).tw. [OBSERVATIONAL STUDIES – Embase Filter – max specificity, Fraser, 2006] (32075334)

41 exp cohort analysis/ or exp case control study/ or controlled clinical trial/ or pretest posttest control group design/ or static group comparison/ or retrospective study/ or longitudinal study/ or intervention study/ or family study/ or case study/ or time series analysis/ or cross-sectional study/ or comparative study/ or observational study/ or quasi experimental study/ or (((cohort or concurrent or non-concurrent or incidence or follow-up or followup or longitudinal or prospective or retrospective or nonrandom$ or non-random$ or quasi-random$ or quasi-experiment$ or quasirandom$ or quasiexperiment$ or pretest or posttest or pre-test or post-test or "before after" or CBA or ITS or (historical$ adj2 control$) or case-control$ or case-comparison or case-compeer or case-referrent or case-referent or case-base or cross-section$ or prevalence) adj3 (stud$ or design?)) or non-RCT or nRCT or real-world or RWE or regist$ or (interrupted adj2 time adj2 series)).tw,kw,kf. [ADDITIONAL TERMS TO SUPPLEMENT NRS FILTERS] (13746999)

42 or/37-41 [All Study Types (except single-arm)] (40278358)

43 32 and 42 [AKI and UO and SC All Study Types (except single-arm)] (3185)

44 (("single arm" or noncomparativ* or non-comparativ* or noncomparison* or non-comparison*) adj3 (cohort? or study or studies or trial?)).tw,kw,kf. (33830)

45 33 and 44 [AKI and UO and Single-Arm Studies Only] (10)

46 34 and 44 [AKI and SC and Single-Arm Studies Only] (37)

47 43 or 45 or 46 (3218)

48 conference abstract.pt. (4454731)

49 47 not 48 (2319)

50 47 and 48 (899)

51 limit 50 to yr="2020-current" (162)

52 49 or 51 [MOST RECENT 2 YRS CONFERENCE ABSTRACTS RETAINED] (2481)

53 52 use oemezd [EMBASE RECORDS] (1364)

54 Acute Kidney Injury/ or (acute$ adj3 (renal$ or kidney?) adj3 (injur$ or fail$ or insufficien$ or nonsufficien$ or non-sufficien$ or trauma? or harm? or damage?)).tw,kw. [Acute Kidney Injury Terms] (189231)

55 ((Urodynamics/ or Urine/ or Urine Specimen Collection/ or Acute Kidney Injury/ur) and (output? or volume? or amount? or measure$ or quantity or quantities).tw,kf,kw.) or ((urin$ adj2 (output? or volume? or amount? or measure$ or quantity or quantities)) or ((postvoid$ or post-void$ or void$) adj3 residual) or urodynamic$).tw,kw. [Urine Output Terms] (162650)

56 Creatinine/bl, ur or (creatinine$ adj3 (serum or sera or blood$ or plasma?)).tw,kw. [Serum Creatinine Terms] (201910)

57 54 and 55 and 56 [AKI and UO and SC ] (3763)

58 (conference abstract or journal conference abstract).pt. (4648025)

59 57 not 58 (2751)

60 57 and 58 (1012)

61 limit 60 to yr="2020-current" (162)

62 59 or 61 [MOST RECENT 2 YRS CONFERENCE ABSTRACTS RETAINED] (2913)

63 62 use cctr [CENTRAL] (262)

64 23 or 53 or 63 [ALL DATABASES] (2691)

65 limit 64 to yr="2012-current" (1984)

66 Acute Kidney Injury/ or (acute$ adj3 (renal$ or kidney?) adj3 (injur$ or fail$ or insufficien$ or nonsufficien$ or non-sufficien$ or trauma? or harm? or damage?)).tw,kf,kw. [Acute Kidney Injury Terms] (193551)

67 ((Urodynamics/ or Urine/ or Urine Specimen Collection/ or Acute Kidney Injury/ur) and (output? or volume? or amount? or measure$ or quantity or quantities).tw,kf,kw.) or ((urin$ adj2 (output? or volume? or amount? or measure$ or quantity or quantities)) or ((postvoid$ or post-void$ or void$) adj3 residual) or urinalys#s or (urin* adj2 analys#s) or urodynamic$).tw,kf,kw. [Urine Output Terms] (211115)

68 (Acute Kidney Injury/di and (Acute Kidney Injury/ur or Urinalysis/)) or ((acute$ adj3 (renal$ or kidney?) adj3 diagnos$).tw,kf,kw. and Urinalysis/) or (acute$ adj3 (renal$ or kidney?) adj3 diagnos$ adj5 urin$).tw,kf,kw. [AKI - DIAGNOSIS - URINALYSIS] (1231)

69 66 and (67 or 68) [AKI and UO/DIAGNOSIS BY URINALYSIS] (10600)

70 (systematic review or systematic literature review or systematic scoping review or systematic narrative review or systematic qualitative review or systematic evidence review or systematic quantitative review or "systematic meta-review" or systematic critical review or systematic mixed studies review or systematic mapping review or systematic cochrane review or "systematic search and review" or systematic integrative review).ti. not comment.pt. not (protocol or protocols).ti. not MEDLINE.st. (258360)

71 (1469-493X or 1361-6137).is. and review.pt. (31651)

72 systematic review.pt. (209441)

73 or/70-72 [Ovid Expert Searches: SLR filter 2019] (482940)

74 (meta-analy$ or metanaly$ or metaanaly$ or met-analy$).mp,pt. or review.pt. [SLR & MA - modified; Montori, 2004 - Balanced query, sn>sp Filter ] (6397716)

75 Network Meta-Analysis/ or ((network adj (MA or MAs)) or (NMA or NMAs or MTC or MTCs or MAIC or MAICs) or indirect$ compar$ or (indirect treatment$ adj1 compar$) or (mixed treatment$ adj1 compar$) or (multiple treatment$ adj1 compar$) or (multi-treatment$ adj1 compar$) or simultaneous$ compar$ or mixed comparison?).tw,kw,kf. [Additional terms for MA, NMA, ITC] (40048)

76 (cochrane or health technology assessment or evidence report or systematic reviews).jw. (67470)

77 (systematic overview$ or evidence-based review$ or evidence-based overview$ or (evidence adj3 (review$ or overview$)) or meta-review$ or meta-overview$ or meta-synthes$ or rapid review$ or "review of reviews" or umbrella review? or technology assessment$ or HTA or HTAs).tw,kw,kf. [Additional terms for synonyms for systematic reviews and HTAs based on SLRs] (181083)

78 or/73-77 [SLR & MA FILTERS - Combined] (6582033)

79 69 and 78 [AKI and UO and SC - SLRs/MAs] (1003)

80 exp Animals/ not (exp Animals/ and Humans/) [ANIMAL STUDIES ONLY - REMOVE - MEDLINE] (17089654)

81 (address or autobiography or bibliography or biography or comment or dictionary or directory or editorial or "expression of concern" or festschrift or historical article or interactive tutorial or lecture or legal case or legislation or news or newspaper article or patient education handout or personal narrative or portrait or video-audio media or webcast or (letter not (letter and randomized controlled trial))).pt. [Opinion publications - Remove -MEDLINE] (4688108)

82 79 not (80 or 81) [ANIMAL-ONLY AND OPINION PIECES REMOVED] (839)

83 82 use ppez [MEDLINE RECORDS] (489)

84 acute kidney failure/ or (acute$ adj3 (renal$ or kidney?) adj3 (injur$ or fail$ or insufficien$ or nonsufficien$ or non-sufficien$ or trauma? or harm? or damage?)).tw,kf,kw. [Acute Kidney Injury Terms] (214500)

85 urine sampling/ or ((urodynamics/ or urine/) and (output? or volume? or amount? or measure$ or quantity or quantities).tw,kf,kw.) or ((urin$ adj2 (output? or volume? or amount? or measure$ or quantity or quantities)) or ((postvoid$ or post-void$ or void$) adj3 residual) or urodynamic$).tw,kf,kw. [Urine Output Terms] (174332)

86 (acute kidney failure/di and exp urinalysis/) or ((acute$ adj3 (renal$ or kidney?) adj3 diagnos$).tw,kf,kw. and exp urinalysis/) or (acute$ adj3 (renal$ or kidney?) adj3 diagnos$ adj5 urin$).tw,kf,kw. [AKI - DIAGNOSIS - URINALYSIS] (1284)

87 84 and (85 or 86) [AKI and UO/DIAGNOSIS - URINALYSIS] (9176)

88 exp Meta Analysis/ or ((meta adj analy$) or metaanalys$).mp. or (systematic adj (review$1 or overview$1)).tw. or (cancerlit or cochrane or embase or psychlit or psyclit or psychinfo or psycinfo or cinahl or cinhal or science citation index or bids or reference lists or bibliograph$ or hand-search$ or manual search$ or relevant journals).ab. (1128769)

89 (data extraction or selection criteria).ab. and review.pt. (67572)

90 88 or 89 [SLR & MA FILTER - Ovid Expert Searches: SLR filter 2019] (1139742)

91 (meta-analy$ or metanaly$ or metaanaly$ or met-analy$).mp. or review.pt. [SLR & MA FILTER - modified and translated; Montori, 2004 - Balanced query, sn>sp Filter ] (6397513)

92 Network Meta-Analysis/ or ((network adj (MA or MAs)) or (NMA or NMAs or MTC or MTCs or MAIC or MAICs) or indirect$ compar$ or (indirect treatment$ adj1 compar$) or (mixed treatment$ adj1 compar$) or (multiple treatment$ adj1 compar$) or (multi-treatment$ adj1 compar$) or simultaneous$ compar$ or mixed comparison?).tw,kf,kw. [Additional terms for MA, NMA, ITC] (40048)

93 (cochrane or health technology assessment or evidence report or systematic reviews).jw. (67470)

94 (systematic overview$ or evidence-based review$ or evidence-based overview$ or (evidence adj3 (review$ or overview$)) or meta-review$ or meta-overview$ or meta-synthes$ or rapid review$ or "review of reviews" or umbrella review? or technology assessment$ or HTA or HTAs).tw,kf,kw. [Additional terms for synonyms for systematic reviews and HTAs based on SLRs] (181083)

95 or/90-94 [SLR & MA FILTERS - Combined] (6705398)

96 87 and 95 [AKI and UO and SC - SRs/MAs] (903)

97 (exp animal/ or exp animal experimentation/ or exp animal model/ or exp animal experiment/ or nonhuman/ or exp vertebrate/) not (exp human/ or exp human experimentation/ or exp human experiment/) [ANIMAL STUDIES ONLY - REMOVE - EMBASE] (11978959)

98 (editorial or letter).pt. not randomized controlled trial/ [OPINION PIECES REMOVE - Embase] (3751379)

99 96 not (97 or 98) [ANIMAL-ONLY AND OPINION PIECES REMOVED] (880)

100 conference abstract.pt. (4454731)

101 99 not 100 (838)

102 99 and 100 (42)

103 limit 102 to yr="2020-current" (10)

104 101 or 103 [MOST RECENT 2 YRS CONFERENCE ABSTRACTS RETAINED] (848)

105 104 use oemezd [EMBASE RECORDS] (486)

106 (acute$ adj3 (renal$ or kidney?) adj3 (injur$ or fail$ or insufficien$ or nonsufficien$ or non-sufficien$ or trauma? or harm? or damage?)).tw,kw. [Acute Kidney Injury Terms] (158166)

107 ((urin$ adj2 (output? or volume? or amount? or measure$ or quantity or quantities)) or ((postvoid$ or post-void$ or void$) adj3 residual) or urodynamic$).tw,kw. [Urine Output Terms] (123500)

108 (acute$ adj3 (renal$ or kidney?) adj3 diagnos$ adj5 urin$).tw,kw. [AKI - DIAGNOSIS - URINALYSIS] (177)

109 106 and (107 or 108) [AKI and UO/DIAGNOSIS - URINALYSIS] (6419)

110 109 use coch [CDSR RECORDS] (66)

111 83 or 105 or 110 [ALL DATABASES] (1041)

112 limit 111 to yr="2012-current" (683)

113 65 or 112 [TRIALS, REVIEWS] (2456)

114 remove duplicates from 113 (1771) [TOTAL UNIQUE RECORDS]

115 114 use ppez [MEDLINE UNIQUE RECORDS] (950)

116 114 use oemezd [EMBASE UNIQUE RECORDS] (643)

117 114 use cctr [CENTRAL UNIQUE RECORDS] (135)

118 114 use coch [CDSR UNIQUE RECORDS] (43)

***************************

1. Press Checklist

***PRESS Guideline* 2015— Search Submission & Peer Review Assessment**

Reference: McGowan J, Sampson M, Salzwedel DM, Cogo E, Foerster V, Lefebvre C. PRESS Peer Review of Electronic Search Strategies: 2015 guideline statement. *J Clin Epidemiol* 2016;75:40-6. Available: <http://www.jclinepi.com/article/S0895-4356(16)00058-5/pdf>.

**Search submission: This section to be filled in by the searcher**

Searcher: Becky Skidmore Email: [becky.skidmore.rls@gmail.com](mailto:becky.skidmore.rls@gmail.com)

Date submitted: 30 Jun 2022 Date requested by: 2-3 Jul 2022

| 1. **Systematic Review Title** |  |
| --- | --- |

Urine output as measure of AKI + including comparisons to serum creatinine as measure of AKI

| 1. **This search strategy is …** |
| --- |

| X | My PRIMARY (core) database strategy — First time submitting a strategy for search question and database |
| --- | --- |
|  | My PRIMARY (core) strategy — Follow-up review NOT the first time submitting a strategy for search question and database. If this is a response to peer review, itemize the changes made to the review suggestions |
|  | SECONDARY search strategy— First time submitting a strategy for search question and database |
|  | SECONDARY search strategy — NOT the first time submitting a strategy for search question and database. If  this is a response to peer review, itemize the changes made to the review suggestions |

| 1. **Database** (e.g., MEDLINE, CINAHL) *[mandatory]* |
| --- |

MEDLINE

| 1. **Interface** (e.g., Ovid, EbscoHost…) *[mandatory]* |
| --- |

Ovid

| 1. **Research Question** (Describe the purpose of the search)  *[mandatory]* |
| --- |

Same as project title

| 1. **PICO Format** Outline the PICOs for your question — i.e., Patient, Intervention, Comparison, Outcome, and Study Design — as applicable |
| --- |

| **P** | Patients assessed for acute kidney injury (AKI) in acute care setting |
| --- | --- |
| **I / Exposure** | Urine output (as indicator of AKI) **note: study does not have to be comparative – can be single-arm study of urine output* |
| **C** | Serum creatinine (as indicator of AKI) **note: study does not have to be comparative – can be single-arm study of serum creatinine* |
| **O** | Accuracy of detection, prediction, and timing of acute kidney injury |
| **S** | RCTs, clinical studies, observational studies, single-arm studies of UO or SC, comparative studies of UO versus SC, SLR and meta-analyses |

| 1. **Inclusion Criteria** (List criteria such as age groups, study designs, etc., to be included) *[optional]*   **This search strategy is …** |
| --- |

| 1. **Exclusion Criteria** (List criteria such as study designs, date limits, etc., to be excluded) **[optional]** |
| --- |

Opinion pieces

For conference abstracts: only include last 2 years

| 1. **Was a search filter applied?** Yes   In-h |
| --- |

**If YES, which one(s) (e.g., Cochrane RCT filter, PubMed Clinical Queries filter)? Provide the source if this is a published filter.** *[mandatory if YES to previous question* — *textbox]*

**Box 3.d** Cochrane Highly Sensitive Search Strategy for identifying randomized trials in MEDLINE: sensitivity- and precision-maximizing version (2008 revision); Ovid format <https://training.cochrane.org/handbook/current/chapter-04-technical-supplement-searching-and-selecting-studies#section-3-6-1>

Furlan AD, Irvin E, Bombardier C. [Limited search strategies were effective in finding relevant nonrandomized studies.](http://www.ncbi.nlm.nih.gov/pubmed/17098573) *J Clin Epidemiol.* 2006;59(12):1303-11

Fraser C, Murray A, Burr J. [Identifying observational studies of surgical interventions in MEDLINE and EMBASE](http://www.biomedcentral.com/1471-2288/6/41). *BMC Med Res Methodol.* 2006;6(41).

| 1. **Notes or comments you feel would be useful for the peer reviewer**  *[optional]* |
| --- |

Team has confirmed:

Single arm SC for detecting AKI

Single arm UO for detecting AKI

Comparative / multi-arm studies between SC and UO for detecting AKI

| 1. **Please copy and paste your search strategy here, exactly as run, including the number of hits per line. [mandatory]** |
| --- |

Database: All Ovid MEDLINE(R) <1946 to Present>

Search Strategy:

--------------------------------------------------------------------------------

1 Acute Kidney Injury/ or (acute$ adj3 (renal$ or kidney?) adj3 (injur$ or fail$ or insufficien$ or nonsufficien$ or non-sufficien$ or trauma? or harm? or damage?)).tw,kf,kw. [Acute Kidney Injury Terms] (78676)

2 ((Urodynamics/ or Urine/) and (output? or volume? or amount? or measure$ or quantity or quantities).tw,kf,kw.) or ((urin$ adj2 (output? or volume? or amount? or measure$ or quantity or quantities)) or ((postvoid$ or post-void$ or void$) adj3 residual) or urodynamic$).tw,kf,kw. [Urine Output Terms] (50341)

3 Creatinine/bl, ur or (creatinine$ adj3 (serum or sera or blood$ or plasma?)).tw,kf,kw. [Serum Creatinine Terms] (90345)

4 1 and 2 and 3 [AKI and UO and SC ] (1179)

5 1 and 2 [AKI and UO] (2333)

6 1 and 3 [AKI and SC] (12194)

7 exp Animals/ not (exp Animals/ and Humans/) [ANIMAL STUDIES ONLY - REMOVE - MEDLINE] (5022766)

8 (address or autobiography or bibliography or biography or comment or dictionary or directory or editorial or "expression of concern" or festschrift or historical article or interactive tutorial or lecture or legal case or legislation or news or newspaper article or patient education handout or personal narrative or portrait or video-audio media or webcast or (letter not (letter and randomized controlled trial))).pt. [Opinion publications - Remove -MEDLINE] (2719864)

9 4 not (7 or 8) [AKI and UO and SC - Animals and Opinion Publications - removed] (1046)

10 5 not (7 or 8) [AKI and UO - Animals and Opinion Publications - removed] (2004)

11 6 not (7 or 8) [AKI and SC - Animals and Opinion Publications - removed] (9625)

12 (randomized controlled trial or controlled clinical trial).pt. or (randomized or placebo or randomly or trial or groups).ab. or drug therapy.fs. [RCTs – MEDLINE sensitive Filter – Cochrane HSSS, 2019] (5393958)

13 exp Randomized Controlled Trials as Topic/ or Clinical Trial, Phase II/ or Clinical Trial, Phase III/ or (equivalence trial or pragmatic clinical trial).pt. or (randomised or randomi#ation? or RCT or placebo$ or ((singl$ or doubl$ or trebl$ or tripl$) adj (mask$ or blind$ or dumm$)) or ((study or trial or CT) adj3 (phase 2 or phase 2a or phase 2b or phase 2c or phase II or phase IIa or phase IIb or phase IIc or phase 3 or phase 3a or phase 3b or phase 3c or phase III or phase IIIa or phase IIIb or phase IIIc or "phase? 2/3" or "phase? II/III")) or open label$).tw,kf. [PHASE 2-3, OPEN LABEL - ADDITIONAL TERMS TO SUPPLEMENT RCTs FILTER] (652322)

14 Cohort studies/ or comparative study/ or follow-up studies/ or prospective studies/ or risk factors/ or cohort.mp. or compared.mp. or groups.mp. or multivariate.mp. [NON-RANDOMIZED STUDIES – MEDLINE Filter - sensitive, Furlan,2006] (8492497)

15 Comparative study/ or Follow-up studies/ or Time factors/ or (preoperat$ or pre operat$).mp. or (chang$ or evaluat$ or reviewed or prospective$ or retrospective$ or baseline or cohort or case series).tw. [OBSERVATIONAL STUDIES – MEDLINE Filter – max specificity, Fraser, 2006] (10861118)

16 non-randomized controlled trials as topic/ or controlled before-after studies/ or interrupted time series analysis/ or historically controlled study/ or case-control studies/ or cross-sectional studies/ or observational study/ or (((cohort or concurrent or non-concurrent or incidence or follow-up or followup or longitudinal or prospective or retrospective or nonrandom$ or non-random$ or quasi-random$ or quasi-experiment$ or quasirandom$ or quasiexperiment$ or pretest or posttest or pre-test or post-test or "before after" or CBA or ITS or (historical$ adj2 control$) or case-control$ or case-comparison or case-compeer or case-referrent or case-referent or case-base or cross-section$ or prevalence) adj3 (stud$ or design?)) or non-RCT or nRCT or real-world or RWE or regist$ or (interrupted adj2 time adj2 series)).tw,kf. [ADDITIONAL TERMS TO SUPPLEMENT NRS FILTERS] (2522549)

17 12 or 13 or 14 or 15 or 16 [All Study Types (except single-arm)] (15538076)

18 9 and 17 [AKI and UO and SC All Study Types (except single-arm)] (890)

19 ("single arm" adj2 (stud$ or design?)).tw,kf. [SINGLE-ARM STUDIES – MEDLINE] (3897)

20 10 and 19 [AKI and UO and Single-Arm Studies Only] (2)

21 11 and 19 [AKI and SC and Single-Arm Studies Only] (5)

22 18 or 20 or 21 (893)

***************************

**Peer review assessment: this section to be filled in by the reviewer**

|  | Reviewer: Kaitryn Campbell | Email: [kcamlolo668@gmail.com](mailto:kcamlolo668@gmail.com) | Date completed: 2 Jul 2022 |
| --- | --- | --- | --- |
|  |  |  |  |

Do you wish to be acknowledged? (If yes, the review team will be advised to add an acknowledgement to any publications related to this work). Yes please.

The suggested acknowledgement is “We thank Kaitryn Campbell, MLIS, MSc (St. Joseph’s Healthcare Hamilton/McMaster University) for peer review of the Medline search strategy.”

|  | **1. TRANSLATION** |  | | |  |
| --- | --- | --- | --- | --- | --- |
| A -­‐No revisions |  |  |  |  |  |
| B -­‐ Revision(s) suggested | X |  |  |  |  |
| C -­‐ Revision(s) required |  |  |  |  |  |

If “B” or “C,” please provide an explanation or example:

Line #19 (single-arm study terms): I’m not sure this line alone is adequate to cover the concept of “single-arm studies”, b/c they can also be referred to in many other ways (e.g., single arm trial, non-comparative study, non-comparative cohort, observational study…)—consider adding additional terms

**2. BOOLEAN AND PROXIMITY OPERATORS**

| A -­‐No revisions | X |
| --- | --- |
| B -­‐ Revision(s) suggested |  |
| C -­‐ Revision(s) required |  |

If “B” or “C,” please provide an explanation or example:

**3. SUBJECT HEADINGS**

| A -­‐No revisions |  |
| --- | --- |
| B -­‐ Revision(s) suggested | X |
| C -­‐ Revision(s) required |  |

If “B” or “C,” please provide an explanation or example:

For line/concept #1 AND line/concept #2, consider adding: Acute Kidney Injury / urine

For line/concept #2, consider adding: Urine Specimen Collection/

**4. TEXT WORD SEARCHING**

| A -­‐No revisions |  |
| --- | --- |
| B -­‐ Revision(s)suggested | X |
| C -­‐ Revision(s) required |  |

If “B” or “C,” please provide an explanation or example:

Consider adding .kf,kw. fields to free-text search terms where relevant for all search filters.

**5. SPELLING, SYNTAX, AND LINE NUMBERS**

| A -­‐No revisions | X |
| --- | --- |
| B -­‐ Revision(s)suggested |  |
| C -­‐ Revision(s) required |  |

If “B” or “C,” please provide an explanation or example:

**6. LIMITS AND FILTERS**

| A -­‐No revisions | X |
| --- | --- |
| B -­‐ Revision(s) suggested |  |
| C -­‐ Revision(s) required |  |

If “B” or “C,” please provide an explanation or example:

OVERALL EVALUATION (Note: If one or more “revision required” is noted above, the response below must be “revisions required”.)

| A -­‐No revisions | X |
| --- | --- |
| B -­‐ Revision(s) suggested |  |
| C -­‐ Revision(s) required |  |

Additional comments:

Elegantly done. I’ve made a few suggestions to consider.
