# Supplementary material for: Urine output is an early and strong predictor of acute kidney injury and associated mortality: a systematic literature review of 50 clinical studies
Source: Ann Intensive Care. 2024 Jul 9;14:110. doi: 10.1186/s13613-024-01342-x (PMC11233478; doi:10.1186/s13613-024-01342-x)
Supplement: Supplementary file 3 — Additional file 3. [file 13613_2024_1342_MOESM3_ESM.docx]

**NICE single technology appraisal quality assessment of the included RCT**

| **Study** | **Random sequence generation (selection bias)** | **Allocation concealment (selection bias)** | **Blinding of participants and personnel (performance bias)** | **Blinding of outcome assessment (detection bias)** | **Incomplete outcome data (attrition bias)** | **Selective Reporting (reporting bias)** | **Other bias** |
| --- | --- | --- | --- | --- | --- | --- | --- |
| McCullough et al. 2016[[30]](#_ENREF_37) | Not clear | Not clear | Low | Low | Low | Low | Low |

Abbreviations: NICE = National Institute for Health and Care Excellence; RCTs = randomized controlled trials
