# Supplementary material for: Urine output is an early and strong predictor of acute kidney injury and associated mortality: a systematic literature review of 50 clinical studies
Source: Ann Intensive Care. 2024 Jul 9;14:110. doi: 10.1186/s13613-024-01342-x (PMC11233478; doi:10.1186/s13613-024-01342-x)
Supplement: Supplementary file 4 — Additional file 4. [file 13613_2024_1342_MOESM4_ESM.docx]

**Newcastle-Ottawa Scale (NOS) quality assessments of the included observational studies**

| **Study** | **Representativeness of Exposed Cohort** | **Selection of the Non-Exposed Cohort** | **Ascertainment of Exposure** | **Comparability of the cohorts on the basis of design or analysis** | **Assessment of outcome** | **Was the follow-up long enough for outcomes to occur?** | **Adequacy of follow-up of cohorts** | **Total** |
| --- | --- | --- | --- | --- | --- | --- | --- | --- |
| Koeze et al. 2020[46] | 1 | 1 | 1 | 2 | 1 | 1 | 1 | 8 |
| D'Arienzo et al. 2019[33] | 1 | 1 | 1 | 1 | 1 | 1 | 1 | 7 |
| Jiang et al. 2021[47] | 1 | 1 | 1 | 2 | 1 | 1 | 1 | 8 |
| Willner et al. 2021[21] | 1 | 1 | 1 | 1 | 1 | 1 | 1 | 7 |
| Bianchi et al. 2021[48] | 1 | 1 | 1 | 2 | 1 | 1 | 1 | 8 |
| Vanmassenhove et al. 2021[34] | 1 | 1 | 1 | 1 | 1 | 1 | 1 | 7 |
| Törnblom et al. 2021[64] | 1 | 1 | 1 | 0 | 1 | 1 | 1 | 6 |
| Nikkinen et al. 2021[49] | 1 | 1 | 1 | 2 | 1 | 1 | 1 | 8 |
| Katabi et al. 2021[65] | 1 | 1 | 1 | 0 | 1 | 1 | 1 | 6 |
| Luther et al. 2021[66] | 1 | 1 | 1 | 0 | 1 | 1 | 1 | 6 |
| Priyanka et al. 2021[50] | 1 | 1 | 1 | 2 | 1 | 1 | 1 | 8 |
| Wiersema et al. 2020[67] | 1 | 1 | 1 | 0 | 1 | 1 | 1 | 6 |
| Allen et al. 2020[35] | 1 | 1 | 1 | 1 | 1 | 1 | 1 | 7 |
| Goldani et al. 2020[68] | 1 | 1 | 1 | 0 | 1 | 1 | 1 | 6 |
| Oshomah-Bello et al. 2020[51] | 1 | 1 | 1 | 2 | 1 | 1 | 1 | 8 |
| Joliat et al. 2020[52] | 1 | 1 | 1 | 2 | 1 | 1 | 1 | 8 |
| Kaddourah et al. 2019[69] | 1 | 1 | 1 | 0 | 1 | 1 | 1 | 6 |
| Bressan et al. 2018[36] | 1 | 1 | 1 | 1 | 1 | 1 | 1 | 7 |
| Howitt et al. 2018[53] | 1 | 1 | 1 | 2 | 1 | 1 | 1 | 8 |
| Hessey et al. 2018[54, 55] | 1 | 1 | 1 | 2 | 1 | 1 | 1 | 8 |
| Amathieu et al. 2017[37] | 1 | 1 | 1 | 1 | 1 | 1 | 1 | 7 |
| Jin et al. 2017[17] | 1 | 1 | 1 | 1 | 1 | 1 | 1 | 7 |
| Palermo et al. 2017[70] | 1 | 1 | 1 | 0 | 1 | 1 | 1 | 6 |
| Koeze et al. 2017[4] | 1 | 1 | 1 | 0 | 1 | 1 | 1 | 6 |
| Petäjä et al. 2017[56] | 1 | 1 | 1 | 2 | 1 | 1 | 1 | 8 |
| Engoren et al. 2017[38] | 1 | 1 | 1 | 1 | 1 | 1 | 1 | 7 |
| Quan et al. 2016[57] | 1 | 1 | 1 | 2 | 1 | 1 | 1 | 8 |
| Cordova-Sanchez et al. 2016[39] | 1 | 1 | 1 | 1 | 1 | 1 | 1 | 7 |
| Qin et al. 2016[58] | 1 | 1 | 1 | 2 | 1 | 1 | 1 | 8 |
| Vaara et al. 2016[59] | 1 | 1 | 1 | 2 | 1 | 1 | 1 | 8 |
| Mizota et al. 2016[41] | 1 | 1 | 1 | 1 | 1 | 1 | 1 | 7 |
| Hocine et al. 2016[71] | 1 | 1 | 1 | 0 | 1 | 1 | 1 | 6 |
| Lagny et al. 2015[60] | 1 | 1 | 1 | 2 | 1 | 1 | 1 | 8 |
| Tujjar et al. 2015[61] | 1 | 1 | 1 | 2 | 1 | 1 | 1 | 8 |
| Harris et al. 2015[41] | 1 | 1 | 1 | 1 | 1 | 1 | 1 | 7 |
| Kellum et al. 2015[8] | 1 | 1 | 1 | 1 | 1 | 1 | 1 | 7 |
| Md Ralib et al. 2013[62] | 1 | 1 | 1 | 2 | 1 | 1 | 1 | 8 |
| McIlroy et al. 2013[42] | 1 | 1 | 1 | 1 | 1 | 1 | 1 | 7 |
| Leite et al. 2013[31] | 0 | 1 | 1 | 2 | 1 | 1 | 1 | 7 |
| Chau et al. 2014[63] | 1 | 1 | 1 | 2 | 1 | 1 | 1 | 8 |
| Shacham et al. 2014[72] | 1 | 1 | 1 | 0 | 1 | 1 | 1 | 6 |
| Wlodzimirow et al. 2012[73] | 1 | 1 | 1 | 0 | 1 | 1 | 1 | 6 |
| Sims et al. 2012[74] | 1 | 1 | 1 | 0 | 1 | 1 | 1 | 6 |
| Han et al. 2012[43] | 1 | 1 | 1 | 1 | 1 | 1 | 1 | 7 |
| Vandenberghe et al. 2022[75] | 1 | 1 | 1 | 0 | 1 | 1 | 1 | 6 |
| Tarvasmaki et al. 2018[44] | 1 | 1 | 1 | 1 | 1 | 1 | 1 | 7 |
| Tulgar et al. 2016[32] | 0 | 1 | 1 | 0 | 1 | 1 | 1 | 5 |
| Bouchard et al. 2015[45] | 1 | 1 | 1 | 1 | 1 | 1 | 1 | 7 |

Abbreviations: NOS = Newcastle-Ottawa Scale.
