# Supplementary material for: Urine output is an early and strong predictor of acute kidney injury and associated mortality: a systematic literature review of 50 clinical studies
Source: Ann Intensive Care. 2024 Jul 9;14:110. doi: 10.1186/s13613-024-01342-x (PMC11233478; doi:10.1186/s13613-024-01342-x)
Supplement: Supplementary file 5 — Additional file 5. [file 13613_2024_1342_MOESM5_ESM.docx]

**Detailed breakdown of adjusted mortality risk**

| **Study** | **Adjusted Factors** | **Analysis Type** | **AKI Diagnostic Method** | **Reference Standard** | **Definition** | **Value**  **(95% CI)** | **Statistical Significance** | **Key Takeaways** |
| --- | --- | --- | --- | --- | --- | --- | --- | --- |
| ***Cardiovascular-related*** | | | | | | | | |
| Howitt et al. 2018[53] | Logistic EuroSCORE | HR | SC | Stage 1 AKI_UO_ | Stage 1 AKI 2-year mortality risk | 1.4  (0.7–2.7) | P = 0.29 | Using UO only as reference, mortality risk within the first two years following surgery was greater for AKI_SC and UO_ than that for AKI_UO_, but the smaller difference in mortality risk between AKI_SC_ and AKI_UO_ over the same period was not statistically significant. |
|  |  |  | SC | Stage 2 AKI_UO_ | Stage 2 AKI 2-year mortality risk | 1.5  (0.6–3.5) | P < 0.01 |  |
|  |  |  | UO and SC | Stage 1 AKI_UO_ | Stage 1 AKI 2-year mortality risk | 1.5  (0.6–3.5) | P = 0.40 |  |
|  |  |  | UO and SC | Stage 2 AKI_UO_ | Stage 2 AKI 2-year mortality risk | 3.6  (1.4–9.3) | P < 0.01 |  |
| McIlroy et al. 2013[42] | Excluding unplanned surgical re-exploration within 48h of surgery | OR | SC | No AKI | AKI In-hospital mortality risk | 7.4  (1.2-45.7) | P = 0.03 | AKI_SC_ was associated with a 7.4-fold increase in mortality. In contrast, AKI_UO_48 was not associated with mortality (P = 0.31), while combining AKI_SC_ or AKI_UO_48 as per AKIN recommendations (i.e., AKI_UO or SC_) also provided no association with mortality (P = 0.44). Oliguria-12 was associated with a marked increase in mortality (P = 0.01), while combining Oliguria-12 with AKI_SC_ provided the greatest association with mortality (P = 0.02). |
|  |  |  | UO (48 h average) | No AKI | AKI In-hospital mortality risk | 3.1  (0.3-28.4) | P = 0.31 |  |
|  |  |  | UO (24 h average) | No AKI | AKI In-hospital mortality risk | 1.9  (0.3-11.9) | P = 0.47 |  |
|  |  |  | UO (12 h average) | No AKI | AKI In-hospital mortality risk | 10.4  (1.6-66.1) | P = 0.01 |  |
|  |  |  | UO (48 h average) and SC | No AKI | AKI In-hospital mortality risk | 2.4  (0.3-21.5) | P = 0.44 |  |
|  |  |  | UO (24 h average) and SC | No AKI | AKI In-hospital mortality risk | 7.4  (0.8-66.6) | P = 0.08 |  |
|  |  |  | UO (12 h average) and SC | No AKI | AKI In-hospital mortality risk | 15.2  (1.7-139) | P = 0.02 |  |
| Tarvasmaki et al. 2018[44] | Age, gender | OR | SC | No AKI | AKI 90-day mortality risk | 7.3  (3.3–16.4) | P<0.001 | After adjustment for different variable sets, AKI_SC_ was consistently associated with increased 90-day mortality. AKI_UO_ was not independently associated with death at 90 days and had no additive value in 90-day mortality prediction compared with AKI_SC_ alone. The stricter AKI_UO_ threshold (i.e., UO <0.3 mL/kg/h for 6 h) showed better discriminative capability for death at 90 days than *<*0.5 mL/kg/h for 6 h. AKI_UO_ with the stricter threshold also retained an independent association with 90-day mortality after multivariable adjustment. |
|  |  |  | UO | No AKI | AKI 90-day mortality risk | 1.7  (0.8–3.4) | P = 0.15 |  |
|  |  |  | UO (*<* 0.3 mL/kg/h for 6 h) | No AKI | AKI 90-day mortality risk | 3.7 (  1.7–8.0) | P = 0.001 |  |
|  | Age, gender, systolic blood pressure, estimated glomerular filtration rate | OR | SC | No AKI | AKI 90-day mortality risk | 7.5  (3.2–17.8) | P<0.001 |  |
|  |  |  | UO | No AKI | AKI 90-day mortality risk | 1.6  (0.8–3.5) | P = 0.2 |  |
|  |  |  | UO (*<* 0.3 mL/kg/h for 6 h) | No AKI | AKI 90-day mortality risk | 3.9  (1.7–9.0) | P = 0.001 |  |
|  | Systolic blood pressure, gender, CardShock risk score (as a continuous variable; includes age and estimated glomerular filtration rate as variables) | OR | SC | No AKI | AKI 90-day mortality risk | 12.2  (4.1–36.0) | P<0.001 |  |
|  |  |  | UO | No AKI | AKI 90-day mortality risk | 1.5  (0.6–3.5) | P = 0.4 |  |
|  |  |  | UO (*<* 0.3 mL/kg/h for 6 h) | No AKI | AKI 90-day mortality risk | 3.6  (1.4–9.3) | P = 0.008 |  |
| Engoren et al. 2017[38] | Demographics, comorbidities, baseline creatinine, and type of operation | OR | UO | No AKI | AKI mortality risk | See Figure 2 | See Figure 2 | Lower UO thresholds for longer times were significantly associated with death (Fig 2). Results were similar for UO being continuously below the threshold for all hours in the time period and for the UO averaged over the time period being below the threshold. |
| Petäjä et al. 2017[56] | EuroSCORE II, BMI, lowest perioperative hematocrit value, vasopressor load, use of inodilators, and postoperative furosemide | HR | UO | No AKI | AKI 2.5-year mortality risk | 3.2  (1.4-7.4) | NR | Mortality among the AKI_SC_, AKI_UO_, and with AKI_UO and SC_ groups was higher than in those without AKI, but mortality did not differ among the AKI groups. |
|  |  |  | SC | No AKI | AKI 2.5-year mortality risk | 3.4  (1.6-7.2) | NR |  |
|  |  |  | UO and SC | No AKI | AKI 2.5-year mortality risk | 4.9  (2.4-9.9) | NR |  |
| ***General ICU*** | | | | | | | | |
| Bianchi et al. 2021[48] | SC stage, age, baseline SC level, Charlson Comorbidity Index, modified SAPS II score, and ICU diagnosis | OR | UO | No AKI | Stage 1 AKI 90-day mortality risk | 1.31 (0.76 - 2.26) | P = .32 | Using no AKI as reference, AKI_UO_ stage 1 was not associated with an increased 90-day mortality. However, AKI_UO_ stage 2 and AKI_UO_ stage 3 were both associated with an increased 90-day mortality. |
|  |  |  | UO | No AKI | Stage 2 AKI 90-day mortality risk | 2.43 (1.57 - 3.77) | P < .001 |  |
|  |  |  | UO | No AKI | Stage 3 AKI 90-day mortality risk | 6.24 (3.69 - 10.52) | P < .001 |  |
| Vanmassenhove et al. 2021[34] | Gender, age, and SOFA score at ICU admission | HR | SC | No AKI stage ≥ 2 | AKI stage ≥ 2 ICU mortality risk | 1.81 (1.56–2.09) | NR | Using no AKI stage ≥ 2 as reference, the UO criterion resulted in relatively higher relative hazards, as compared to only considering the SC criterion. This pattern was found irrespective of whether the UO criterion was (1) strictly interpreted (UO-2) or more broadly (UO-1), (2) considered in combination with SC or not, or (3) adjusted for other risk factors or not. |
|  |  |  | UO-1 | No AKI stage ≥ 2 | AKI stage ≥ 2 ICU mortality risk | 2.59 (2.18–3.09) | NR |  |
|  |  |  | UO-2 | No AKI stage ≥ 2 | AKI stage ≥ 2 ICU mortality risk | 2.83 (2.44–3.28) | NR |  |
|  |  |  | UO or SC-1 | No AKI stage ≥ 2 | AKI stage ≥ 2 ICU mortality risk | 2.54 (2.14–3.02) | NR |  |
|  |  |  | UO or SC-2 | No AKI stage ≥ 2 | AKI stage ≥ 2 ICU mortality risk | 2.62 (2.27–3.04) | NR |  |
| Vaara et al. 2016[59] | Age, sex, APACHE II diagnosis group, SAPS II score without age and renal components, use of vasoactives, diuretics, or RRT, and the cumulative fluid balance (% of baseline weight) on the day of oliguric episode | OR | UO (0.3 to <0.5 ml/kg/h) for 3-6h | No oliguria^a^ / oliguria lasting <3h | 90-day morality risk | 0.96 (0.68–1.37) | P = 0.83 | The shortest periods of consecutive oliguria independently associated with an increased risk for 90-day mortality were 6–12 h of oliguria from 0.3 to <0.5 ml/kg/h, over 6 h of oliguria from 0.1 to <0.3 ml/kg/h, and severe oliguria lasting over 3 h. |
|  |  |  | UO (0.3 to <0.5 ml/kg/h) for <6h | No oliguria^a^ / oliguria lasting <3h | 90-day morality risk | 1.65 (1.00–2.72) | P = 0.05 |  |
|  |  |  | UO (0.1 to <0.3 ml/kg/h) for 3-6h | No oliguria^a^ / oliguria lasting <3h | 90-day morality risk | 1.20 (0.87–1.65) | P = 0.28 |  |
|  |  |  | UO (0.1 to <0.3 ml/kg/h) for <6h | No oliguria^a^ / oliguria lasting <3h | 90-day morality risk | 1.96 (1.13–3.38) | P = 0.02 |  |
|  |  |  | UO (<0.1 ml/kg/h) for 3-6h | No oliguria^a^ / oliguria lasting <3h | 90-day morality risk | 2.08 (1.27–3.42) | P = 0.01 |  |
|  |  |  | UO (<0.1 ml/kg/h) for 6-12h | No oliguria^a^ / oliguria lasting <3h | 90-day morality risk | 3.04 (1.50–6.15) | P = 0.01 |  |
|  |  |  | UO (<0.1 ml/kg/h) for 12-24h | No oliguria^a^ / oliguria lasting <3h | 90-day morality risk | 6.78 (2.79–16.44) | P<0.001 |  |
|  |  |  | UO (<0.1 ml/kg/h) for >24h | No oliguria^a^ / oliguria lasting <3h | 90-day morality risk | 9.76 (4.07–23.43) | P<0.001 |  |
| Harris 2015[^56^](#_ENREF_56) | Complementary RIFLE component (i.e., SC adjusted for UO; UO adjusted for SC) | RR | SC | No AKI (0.75-1.25 ∆SC) | No AKI (≤0.4 ∆SC) ICU mortality risk | 0.9 (0.8–1.0) | NR | UO is a more potent predictor of ICU mortality compared with SC partly because mortality fell when peak/estimated baseline creatinine ratios exceed fourfold. The effect is robust to adjustment for the complementary component of RIFLE class. |
|  |  |  | SC | No AKI (0.75-1.25 ∆SC) | No AKI (0.4-0.75 ∆SC) ICU mortality risk | 0.7 (0.7–0.7) | NR |  |
|  |  |  | SC | No AKI (0.75-1.25 ∆SC) | No AKI (0-75-1.25 ∆SC) ICU mortality risk | 1.5 (1.4–1.5) | NR |  |
|  |  |  | SC | No AKI (0.75-1.25 ∆SC) | AKI-Risk (1.5-2 ∆SC) ICU mortality risk | 1.7 (1.7–1.8) | NR |  |
|  |  |  | SC | No AKI (0.75-1.25 ∆SC) | AKI-Injury (2-3 ∆SC) ICU mortality risk | 1.7 (1.7–1.8) | NR |  |
|  |  |  | SC | No AKI (0.75-1.25 ∆SC) | AKI-Failure (3-4 ∆SC) ICU mortality risk | 1.5 (1.5–1.6) | NR |  |
|  |  |  | SC | No AKI (0.75-1.25 ∆SC) | AKI-Failure (4-6 ∆SC) ICU mortality risk | 1.3 (1.2–1.3) | NR |  |
|  |  |  | SC | No AKI (0.75-1.25 ∆SC) | AKI-Failure (≥ 6 ∆SC) ICU mortality risk | 1.0 (0.9–1.0) | NR |  |
|  | Complementary RIFLE component (i.e., SC adjusted for UO; UO adjusted for SC) | RR | UO | AKI-Failure (2000-5000 ml/24 h) | No AKI (≥5000ml/24 h) ICU mortality risk | 1.6 (1.6–1.7) | NR |  |
|  |  |  | UO | AKI-Failure (2000-5000 ml/24 h) | AKI-Failure (1500-2000 ml/24 h) ICU mortality risk | 1.2 (1.1–1.2) | NR |  |
|  |  |  | UO | AKI-Failure (2000-5000 ml/24 h) | AKI-Failure (800-1500 ml/24 h) ICU mortality risk | 1.6 (1.5–1.6) | NR |  |
|  |  |  | UO | AKI-Failure (2000-5000 ml/24 h) | AKI-Risk (500-850ml/24 h) ICU mortality risk | 2.3 (2.2–2.4) | NR |  |
|  |  |  | UO | AKI-Failure (2000-5000 ml/24 h) | AKI-Injury (100-500ml/24 h) ICU mortality risk | 3.5 (3.4–3.6) | NR |  |
|  |  |  | UO | AKI-Failure (2000-5000 ml/24 h) | AKI-Failure (0-100ml/24 h) ICU mortality risk | 4.7 (4.5–4.9) | NR |  |
| Han et al. 2012[43] | Diuretic dose | HR | SC | No AKI | Stage 1 AKI overall mortality risk | 1.75 (1.52–2.02) | NR | The dose of diuretics did not alter the relationship between AKI staging and mortality; the unadjusted HRs for diuretic dose were similar to the  adjusted HRs. |
|  |  |  |  | No AKI | Stage 2 AKI overall mortality risk | 2.93 (2.30–3.72) | NR |  |
|  |  |  |  | No AKI | Stage 3 AKI overall mortality risk | 3.23 (2.66–3.93) | NR |  |
|  |  |  | UO | No AKI | Stage 1 AKI overall mortality risk | 1.81 (1.51–2.16) | NR |  |
|  |  |  |  | No AKI | Stage 2 AKI overall mortality risk | 2.96 (2.38–3.68) | NR |  |
|  |  |  |  | No AKI | Stage 3 AKI overall mortality risk | 4.17 (3.31–5.26) | NR |  |
|  |  |  | UO and SC | No AKI | Stage 1 AKI overall mortality risk | 1.67 (1.44–1.94) | NR |  |
|  |  |  |  | No AKI | Stage 2 AKI overall mortality risk | 3.19 (2.58–3.94) | NR |  |
|  |  |  |  | No AKI | Stage 3 AKI overall mortality risk | 3.57 (2.94–4.33) | NR |  |
| Qin et al. 2016[58] | Gender, age, APACHE II score, SOFA score, comorbidities, admission status, reasons of ICU admission, renal function on ICU admission, complications | OR | UO | No AKI | AKI Hospital mortality risk | 2.89 (1.96–4.25) | P<0.001 | AKI_UO_, but not AKI_SC_, was an independent risk factor for hospital mortality, after adjusting for other potential confounders. |
|  |  |  | SC | No AKI | AKI Hospital mortality risk | 1.32 (0.90–1.94) | P = 0.152 |  |
| Kellum et al. 2015[8] | Age | NR | UO | No AKI | AKI 1-year risk for death or RRT | NR | NR | Short- and long-term risk of death or RRT is greatest when patients meet criteria for AKI_UO and SC_ and when these abnormalities persist (Figure 1, top). Overall, increasing stage is associated with lower survival (P<0.001). However, when AKI_UO_ is present without AKI_SC_, stage 1 does not separate from no AKI (P=0.12). Conversely, with AKI_SC_ and no AKI_UO_, stages 2 and 3 do not separate (P=0.27). |
|  |  |  | SC | No AKI | AKI 1-year risk for death or RRT | NR | NR |  |
|  |  |  | UO or SC | No AKI | AKI 1-year risk for death or RRT | NR | NR |  |
| Jin et al. 2017[17] | Age and APS-III | HR | UO – Intensive | Less intensive, AKI | AKI 30-day mortality risk | 0.90 (0.81-0.99) | p<0.04 | For patients with AKI, intensive monitoring for UO was strongly associated with improved survival to 30 days compared with patients with less intensive UO monitoring. No association was found between intensity of SC monitoring. |
|  |  |  | SC – Intensive | Less intensive, AKI | AKI 30-day mortality risk | 1.10 (0.98-1.24) | p< 0.11 |  |
| ***Pediatric*** | | | | | | | | |
| Hessey et al. 2018a[55] | Age, sex, primary PICU diagnosis of cancer, primary PICU diagnosis of infection, PRISM score death rate, vasopressors, nephrotoxic antibiotics, and steroids received in the PICU and interaction of AKI x age, AKI x cancer diagnosis, and AKI x infection diagnosis | HR | SC | No AKI | AKI 5–7-year morality risk | 3.10 (1.46–6.57) | P <0 .05 | AKI was independently associated with 5- to 7-year mortality. Including UO did not strengthen the association. The association of AKI stage 2 or 3 (versus no AKI or stage 1) with mortality was not stronger than the  association of any AKI with mortality. |
|  |  |  | SC | No AKI or stage 1 AKI | Stage 2 or 3 AKI 5–7-year morality risk | 1.86 (0.91–3.79) | NR |  |
|  |  |  | UO or SC | No AKI | AKI 5–7-year morality risk | 3.38 (1.63–7.02) | P <0 .05 |  |
|  |  |  | UO or SC | No AKI or stage 1 AKI | Stage 2 or 3 AKI 5–7-year morality risk | 1.62 (0.81–3.22) | NR |  |
| ***Kidney-related*** | | | | | | | | |
| Jiang et al. 2021[47] | Group variable, age, sex, and BMI | OR | UO | No AKI | AKI Hospital mortality risk | 2.88 (2.39 - 3.45) | NR | Using non-AKI group as reference, both UO group and SC group were associated with significantly higher hospital and 90-day mortality. |
|  |  |  | UO | No AKI | AKI 90-day mortality risk | 2.07 (1.78 - 2.40) | NR |  |
|  |  | OR | SC | No AKI | AKI Hospital mortality risk | 2.26 (1.88 - 2.7) | NR |  |
|  |  |  | SC | No AKI | AKI 90-day mortality risk | 1.61 (1.39 - 1.86) | NR |  |
| ***Liver-related*** | | | | | | | | |
| Mizota et al. 2016[41] | Age, MELD score > 30, blood type incompatibility and re-transplantation | OR | SC | No AKI | Stage 1 AKI hospital mortality | 0.97 (0.39–2.40 | NR | Hospital mortality among patients with stage 3 AKI_SC_ increased nearly eightfold when compared to those without AKI_SC_, whereas stages 1–2 AKI_SC_ were not significantly associated with an increase in hospital mortality. When AKI_UO and SC_ was used instead of AKI_SC_, stage-wise increase in hospital mortality was observed and stage 2 and stage 3 AKI_UO and SC_ were significantly associated with an increase in hospital mortality. |
|  |  |  | SC | No AKI | Stage 2 AKI hospital mortality | 1.31 (0.46–3.78 | NR |  |
|  |  |  | SC | No AKI | Stage 3 AKI hospital mortality | 7.87 (3.26–19.00) | NR |  |
|  |  |  | UO and SC | No AKI | Stage 1 AKI hospital mortality | 1.16 (0.47–2.90) | NR |  |
|  |  |  | UO and SC | No AKI | Stage 2 AKI hospital mortality | 2.71 (1.14–6.46) | NR |  |
|  |  |  | UO and SC | No AKI | Stage 3 AKI hospital mortality | 8.66 (3.33–22.60) | NR |  |
| Joliat et al. 2020[52] | Gender and BMI | HR | SC | No AKI | AKI 30-day mortality risk | 13.3 (1.1–163.5) | P = 0.044 | Development of AKI_SC_ was found as an independent predictor of 30-day mortality after hepatectomy, whereas AKI_UO_ was not |
|  |  |  | UO | No AKI | AKI 30-day mortality risk | 3.5 (0.3–45.0) | P = 0.336 |  |
| ***Non-Cardiac Surgery*** | | | | | | | | |
| Quan et al. 2016[57] | Sex, age, type of surgery, baseline proteinuria, baseline eGFR, each of the Charlson comorbidities and hypertension | OR | SC | No AKI | Stage 1 AKI 30-day mortality risk | 5.27 (3.05–9.10) | C-statistic: 0.88 | Compared with those without AKI, patients with stage 1, 2, or 3 AKI had higher adjusted 30‑day mortality for AKI_SC_, AKI_UO_, and AKI_UO or SC._ |
|  |  |  | SC | No AKI | Stage 2 AKI 30-day mortality risk | 8.62 (3.14–23.66) |  |  |
|  |  |  | SC | No AKI | Stage 3 AKI 30-day mortality risk | 5.00 (1.65–15.17)^b^ |  |  |
|  |  |  | UO or SC | No AKI | Stage 1 AKI 30-day mortality risk | 1.55 (0.33–7.39) | C-statistic: 0.86 |  |
|  |  |  | UO or SC | No AKI | Stage 2 AKI 30-day mortality risk | 1.75 (0.83–3.68) |  |  |
|  |  |  | UO or SC | No AKI | Stage 3 AKI 30-day mortality risk | 2.84 (1.41–5.70) |  |  |
|  |  |  | UO | No AKI | Stage 1 AKI 30-day mortality risk | 9.90 (2.66–36.80) | C-statistic: 0.88 |  |
|  |  |  | UO | No AKI | Stage 2 AKI 30-day mortality risk | 4.89 (1.67–14.33) |  |  |
|  |  |  | UO | No AKI | Stage 3 AKI 30-day mortality risk | 7.85 (2.76–22.33) |  |  |

^a^ UO <0.5 ml/kg/h

^b^ There was no statistically significant difference in adjusted estimates of 30-day mortality between Stage 2 and Stage 3 AKI incorporating SC criteria (P = 0.745).

Abbreviations: AKI = acute kidney injury; APACHE = Acute Physiology and Chronic Health Evaluation; APS = Acute Physiology Score; BMI = body mass index; CI = confidence interval; eGFR = estimated glomerular filtration rate; EuroSCORE = European System for Cardiac Operative Risk Evaluation; HR = hazard ratio; ICU = intensive care unit; MELD = Model for End-Stage Liver Disease; NR = not reported; OR = odds ratio; PICU = pediatric intensive care unit; PRISM = Pediatric Risk of Mortality Score; RRT = renal replacement therapy; SAPS = Simplified Acute Physiology Score; SC = serum creatinine; SOFA = Sequential Organ Failure Assessment; UO = urine output; UO or SC = patients met AKI criteria by UO, SC, or both; UO and SC = patients met AKI criteria by both UO and SC.
