# Supplementary material for: Urine output is an early and strong predictor of acute kidney injury and associated mortality: a systematic literature review of 50 clinical studies
Source: Ann Intensive Care. 2024 Jul 9;14:110. doi: 10.1186/s13613-024-01342-x (PMC11233478; doi:10.1186/s13613-024-01342-x)
Supplement: Supplementary file 6 — Additional file 6. [file 13613_2024_1342_MOESM6_ESM.docx]

**Adjusted mortality risk in AKI patients versus no AKI patients by AKI stage**


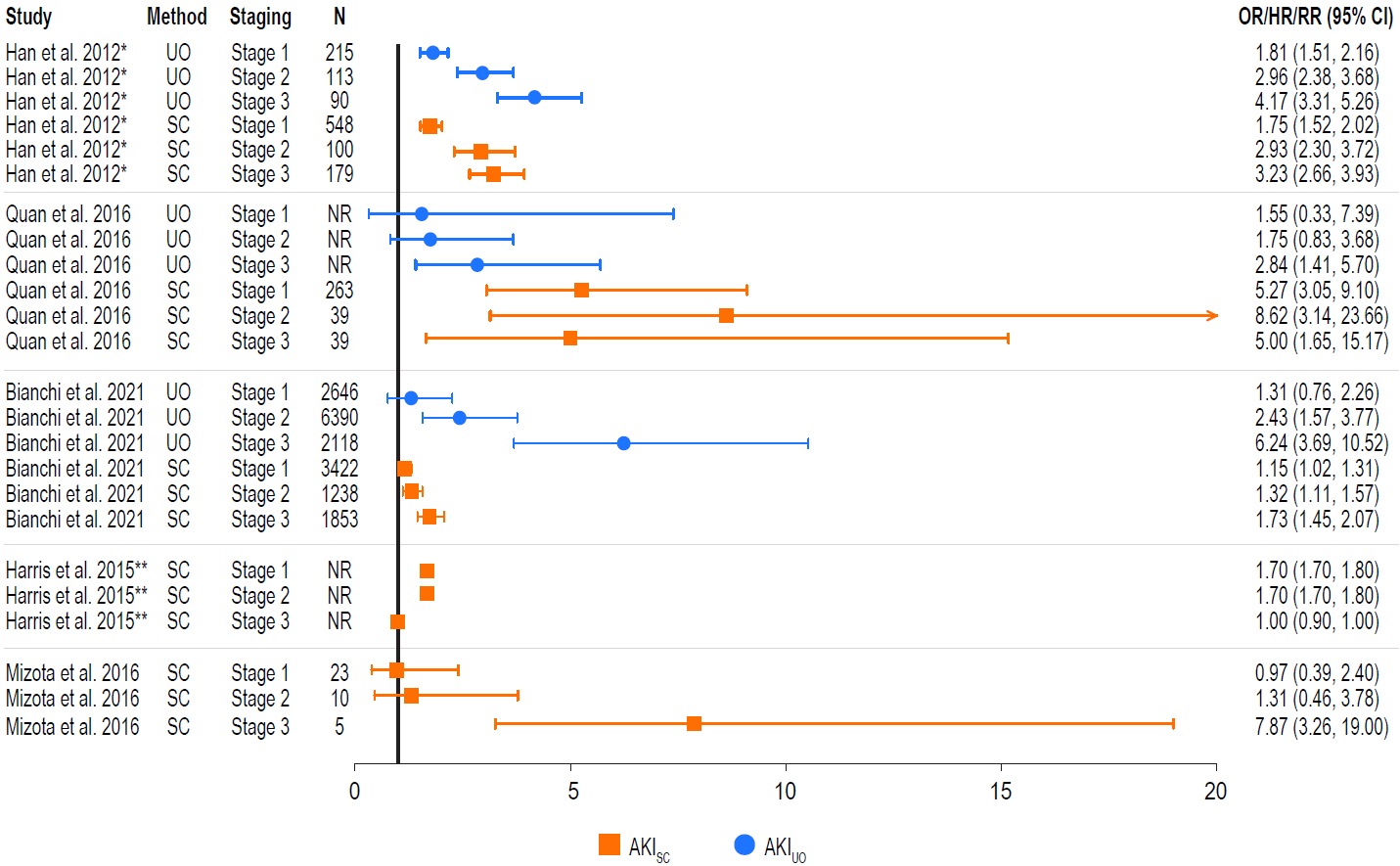


* Data presented as HR (95% CI) where no OR (95% CI) was reported. Error bars correspond to the 95% CI and may extend past visible axis.

** Data presented as RR (95% CI) where no OR (95% CI) was reported. Error bars correspond to the 95% CI and may extend past visible axis.

Boxplots comparing AKI incidence based on diagnostic criteria used. The error bars are the 95% confidence interval, the bottom and top of the box are the 25th and 75th percentiles, the line inside the box is the 50th percentile (median).

Abbreviations: AKI = acute kidney injury; CI = confidence interval; HR = hazard ratio; OR = odds ratio; RR = risk ratio; SC = serum creatinine; UO = urine output.
