# Supplementary material for: Urine output is an early and strong predictor of acute kidney injury and associated mortality: a systematic literature review of 50 clinical studies
Source: Ann Intensive Care. 2024 Jul 9;14:110. doi: 10.1186/s13613-024-01342-x (PMC11233478; doi:10.1186/s13613-024-01342-x)
Supplement: Supplementary file 7 — Additional file 7. [file 13613_2024_1342_MOESM7_ESM.docx]

Hospital length of stay among patients with AKI


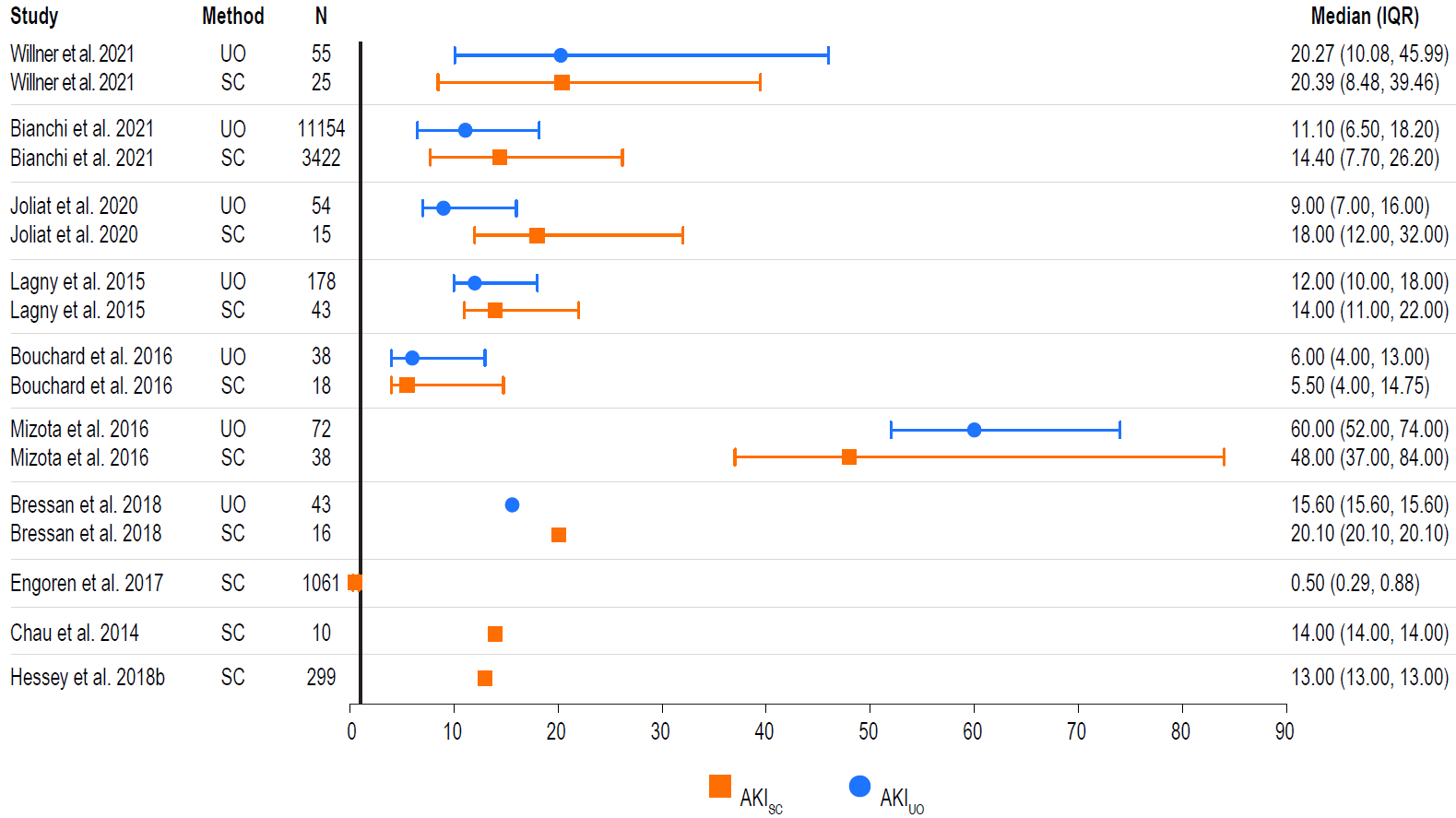


The median ICU length of stay is graphically represented per study and method as a point, with error bars as the IQR. Median values with the associated IQR are listed to the right of the figure. Note that unadjusted results are presented.

Abbreviations: AKI = acute kidney injury; IQR=interquartile range; SC = serum creatinine; UO = urine output.
